# Lipoprotein(a) in Atherosclerotic Diseases: From Pathophysiology to Diagnosis and Treatment

**DOI:** 10.3390/molecules28030969

**Published:** 2023-01-18

**Authors:** Stamatios Lampsas, Maria Xenou, Evangelos Oikonomou, Panteleimon Pantelidis, Antonios Lysandrou, Savvas Sarantos, Athina Goliopoulou, Konstantinos Kalogeras, Vasiliki Tsigkou, Athanasios Kalpis, Stavroula A. Paschou, Panagiotis Theofilis, Manolis Vavuranakis, Dimitris Tousoulis, Gerasimos Siasos

**Affiliations:** 1 3rd Department of Cardiology, National and Kapodistrian University of Athens, Medical School, Sotiria Chest Disease Hospital, 11527 Athens, Greece; 2 1st Department of Cardiology, National and Kapodistrian University of Athens, Medical School, Hippokration General Hospital, 11527 Athens, Greece; 3 Cardiovascular Division, Brigham and Women’s Hospital, Harvard Medical School, Boston, MA 02115, USA

**Keywords:** lipoprotein(a), Lp(a), genetic variations, atherosclerotic disease, cardiovascular disease, thrombosis, inflammation, treatment

## Abstract

Lipoprotein(a) (Lp(a)) is a low-density lipoprotein (LDL) cholesterol-like particle bound to apolipoprotein(a). Increased Lp(a) levels are an independent, heritable causal risk factor for atherosclerotic cardiovascular disease (ASCVD) as they are largely determined by variations in the Lp(a) gene (LPA) locus encoding apo(a). Lp(a) is the preferential lipoprotein carrier for oxidized phospholipids (OxPL), and its role adversely affects vascular inflammation, atherosclerotic lesions, endothelial function and thrombogenicity, which pathophysiologically leads to cardiovascular (CV) events. Despite this crucial role of Lp(a), its measurement lacks a globally unified method, and, between different laboratories, results need standardization. Standard antilipidemic therapies, such as statins, fibrates and ezetimibe, have a mediocre effect on Lp(a) levels, although it is not yet clear whether such treatments can affect CV events and prognosis. This narrative review aims to summarize knowledge regarding the mechanisms mediating the effect of Lp(a) on inflammation, atherosclerosis and thrombosis and discuss current diagnostic and therapeutic potentials.

## 1. Introduction

Atherosclerosis constitutes a chronic arterial disease, initiated very early in life, while its progression appears slow, with its clinical manifestations peaking during the fifth and sixth decades of life [1,2]. Atherosclerosis is the underlying cause of most cardiovascular diseases (CVD), which account for more than 17.9 million deaths per year [3]. Inflammation has a pivotal role in this context as both innate and adaptive immunoinflammatory mechanisms are involved in the atherosclerotic properties of the arterial wall [4]. Atherosclerotic cardiovascular diseases (ASCVD) are highly dependent on modified lifestyle factors, such as obesity, type 2 diabetes mellitus (T2DM) and metabolic syndrome [5]. Moreover, older age, hypertension, dyslipidemias, cigarette-smoking and physical inactivity are some of the traditional risk factors leading to acceleration of atherogenic processes and early cardiovascular (CV) events [5].

However, genetic predisposition is a key factor in ASCVD risk and progression, and, despite effective management of atherosclerotic risk factors, residual atherosclerotic CV risk appears to be significant. Elevated lipoprotein(a) (Lp(a)) in plasma is an independent, mainly genetically determined causal risk factor for CVD [6]. Lp(a) was first described in 1963 by Kåre Berg as a unique lipoprotein similar to low-density lipoprotein (LDL) [7]. Lp(a) transverses the endothelium and accumulates in the subendothelial space, with thrombogenic and atherogenic potential [8,9]. Evolving evidence underlines that high Lp(a) levels incur higher rates of CVD, accelerate progression and increase CV mortality [6,10,11]. Despite the emerging data on the role of Lp(a) in ASCVD, little is known about relevant treatment options. Moreover, there are diagnostic obstacles in the field since standardized Lp(a) measurement methods are lacking [12,13]. Therefore, this review aims to shed light upon the molecular mechanisms mediating the role of Lp(a) in inflammation, atherosclerosis and thrombosis and discuss current diagnostic and therapeutic approaches.

## 2. Structure Variations and Genetics of Lp(a)

### 2.1. Structure of the Lp(a) Particle

**Figure 1:**
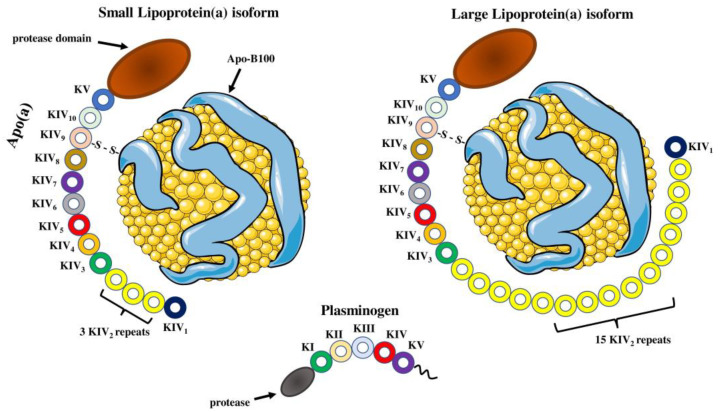
Structure of lipoprotein(a) and plasminogen. Human Lp(a) consists of an LDL-like particle and an apoB-100 particle, in which glycoprotein apo(a) is disulfide-linked. Apo(a) contains 10 different types of plasminogen-like kringle IV domains (KIVs), composed of 1 copy of KIV1, multiple copies of KIV2 and 1 copy of KIV3-10 as well as a single kringle V domain followed by an inactive protease domain. KIV2 repeats of apo(a) determine the size of different Lp(a) isoforms. Apo(a) and plasminogen share high amino acid sequence similarity, including the protease domain and kringles type IV and type V. Parts of the figure were drawn by using pictures from Servier Medical Art. Servier Medical Art by Servier is licensed under a Creative Commons Attribution 3.0 Unported License (https://creativecommons.org/licenses/by/3.0/) (accessed on 4 November 2022).

**Figure 2:**
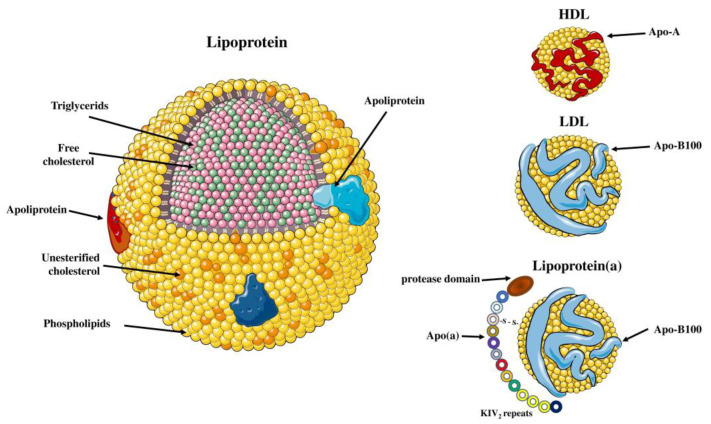
Differences between Lp(a), LDL and HDL. Lipoprotein(a) is composed of one LDL particle containing apo-B100 and apo(a). HDL is composed of a high-density lipid core, and the most common apolipoproteins found on its surface are apo A-I and apo A-II. Parts of the figure were drawn by using pictures from Servier Medical Art. Servier Medical Art by Servier is licensed under a Creative Commons Attribution 3.0 Unported License (https://creativecommons.org/licenses/by/3.0/) (accessed on 4 November 2022).

Lp(a) is a low-density lipoprotein (LDL)-like molecule, composed of a lipid core of cholesteryl esters and triacylglycerols, with an outer shell of phospholipids, free cholesterol and apolipoprotein B-100 (apoB-100) particles [12,14]. However, Lp(a) differs from LDL cholesterol since it includes an additional characteristic glycoprotein, apolipoprotein(a) (apo(a)), which is attached to the apoB-100 by a single disulfide bond (Figure 1) [15,16]. Biosynthesis of Lp(a) takes place almost exclusively in the liver as significant amounts of apo(a) messenger RNA (mRNA) were detected in hepatocytes from humans, baboons and macaques, but also minor amounts were discovered in testes, brain, lung and adrenal and pituitary glands from humans and monkeys [17,18]. This process involves synthesis of apo(a) in hepatocytes, followed by apoB-100 binding [19]. The site of Lp(a) assembly is unclear as three possible sites have been proposed: A. intracellularly; B. on the cell surface; C. extracellularly [20,21]. In vitro studies have revealed that there is no coordination between the synthesis pathways of apo(a) and apoB-100 [22]. In contrast to LDL, the plasma concentration of Lp(a) has little or no relationship with the fractional catabolic rate of Lp(a), but it is highly correlated with the Lp(a) production rate [23,24]. Lp(a) biosynthesis results in assembly of a spherical, macromolecular lipoprotein complex with a diameter of approximately 25 nm and a density ranging from 1.05 to 1.12 g/mL [25]. High-density lipoprotein (HDL), LDL and Lp(a) all have a spherical shape, with HDL having the smallest diameter (7–13 nm), while Lp(a) and LDL have approximately the same size (~25 nm) [26]. The most abundant apolipoproteins in HDL are apolipoprotein A-I (apo A-I) and apolipoprotein A-II (A-II) [27]. Moreover, HDL has greater density than Lp(a) (1.06–1.21 g/mL) as it contains the highest ratio of proteins to lipids [26] (Figure 2).

### 2.2. Apolipoprotein(a) Structure and Function

The pathophysiological function of Lp(a) is mainly attributed to the presence of its apo(a) subunit. The presence of apo(a) determines the difference between LDL and Lp(a) density, electrophoretic mobility and molecular weight as this glycoprotein varies widely from 400 to 700 kDa [28]. Apo(a) has a similar overall structure to plasminogen, one of the proteins of the fibrinolytic system [29]. Apo(a) contains a unique protein domain known as “kringle”, made up of 80 amino acids [30]. Apo(a) comprises multiple plasminogen-like kringle IV (KIV) domains and a single kringle V domain followed by an inactive protease domain located at the carboxyl terminus of the molecule [31]. KIV domain is sub-divided and numbered from type 1–10 based on the differences in amino acid sequence and contains ten different types of KIV1 to KIV10 [32]. All KIV domains are present as single copies, except for kringle IV type 2 (KIV2), which appears in a variable number of copies [33]. The variable number of KIV2 repeats makes it one of the most polymorphic glycoproteins in human plasma [34]. Size heterogeneity of the apo(a) isoforms is due to different numbers of KIV2 repeats coding sequences in the apo(a) gene [35,36]. Studies demonstrated that size of hepatic apo(a) mRNA was correlated with size of apo(a) protein [30,37]. Additionally, the heterogeneity in apo(a) isoforms is also attributed to differences in protein folding, transport and secretion between larger and smaller isoforms [38]. Single nucleotide polymorphisms (SNPs) of the *LPA* gene also play a critical role in apo(a) heterogeneity as they affect RNA splicing, nonsense mutations and the 5′ untranslated region of the apolipoprotein(a) gene, inducing shorter gene translation [39,40]. Much effort has been put into defining the physiological role of apo(a), but its exact mechanism of action has not been determined. Experimental studies demonstrate regulatory action of apo(a) in inflammation and wound healing as well as a modulatory role in the cholesterol efflux capacity of cells [41,42,43].

Moreover, several studies proclaim the detrimental effect of smaller apo(a) isoforms [44]. Smaller apo(a) particles, resulting in smaller Lp(a) molecules, appear to (1) increase the capacity of bound oxidized phospholipids; (2) accumulate more in blood vessel walls through increased lysine-binding ability and interaction with fibrin; (3) increase inhibition of plasmin activity, resulting in greater thrombogenicity [45]. Furthermore, smaller apo(a) isoforms show synergistic action with small-dense LDL and oxidized LDL (OxLDL) particles [46].

### 2.3. LPA Gene Variations and Atherosclerotic Disease

The *LPA* gene, which encodes the apo(a) component of the Lp(a) particle and is located on the long arm of chromosome 6 within 6q2.6–2.7 [47], shows homology up to 70% with the plasminogen gene [29]. The heterogeneity in apo(a), and, consequently, Lp(a) size, is based on the number of KIV2 copies, which play a central role in controlling the circulatory levels of Lp(a) [48,49]. Fewer KIV2 repeats reduce the apo(a) size, leading to higher Lp(a) levels as hepatocytes can produce smaller apo(a) particles at higher rates [50].

The apo(a) isoforms are modified by many SNPs distributed over the entire range of allele frequencies, with very strong effects on Lp(a) concentrations [51]. Genome-wide association studies identify two SNPs in the *LPA* gene, which are very strongly and independently correlated with increased Lp(a) levels and higher risk for CVD [44,52,53,54]: Rs3798220 and rs10455872 SNPs. However, the mechanisms underlining the effect of SNPs on apo(a) size and Lp(a) levels are still vaguely understood. Large studies reveal that *LPA* gene polymorphisms, such as rs783147, rs3798220 and rs10455872, are also strongly associated with CV lesions, such as increased carotid intima-media thickness and impaired endothelial function, suggesting a direct role of *LPA* gene SNPs in early atherosclerotic changes [55].

## 3. Measurement of Plasma Lp(a) Concentration and Standardization

While Lp(a) has started to emerge as a risk factor for cardiovascular disease, with recommendations for measuring it at least once during lifetime, especially in high-risk populations [56,57], issues regarding standardization and validity of methods used to measure it are still present. Since the size of Lp(a) is highly variable and largely determined by the apo(a) size, which varies across different populations, there is inter-individual and intra-individual variability as most individuals are carriers of two different apo(a) alleles [40,58,59]. Accordingly, kits that aim to quantify the mass concentration of Lp(a) are more prone to variation compared to molarity-measuring tests, leading to diverging values of different mass-targeting kits, even within the same population with a standard Lp(a) molar concentration [60,61]. Despite this issue and the apo(a)-insensitive quantifying methods, [61,62] commercial kits measuring Lp(a) in mg/dL instead of estimating its molarity in nmol/L are still amply encountered in practice and the literature [63,64]. Moreover, converting the mass concentration of Lp(a) to its molar equivalent (mg/dL to nmol/L) cannot produce accurate results, although attempts have been made and a rough 2–2.5× conversion factor has been proposed [65].

## 4. Role of Lp(a) on Atherosclerosis

### 4.1. Attachment of Lp(a) to Arterial Wall

Lp(a) exerts its atherogenic actions when transferred from circulation to the arterial wall. In general, the arterial influx of lipoproteins depends on arterial wall permeability, the concentration of lipoprotein in plasma and arterial blood pressure [66]. Lp(a) has been detected in human vessels and is concentrated mainly extracellularly in the intima and subintima [8]. Anchoring of Lp(a) depends on its two components: its lipoproteinic structure and the lysine binding sites of apo(a) [67,68]. Notably, Lp(a) is accumulated in the arterial wall to a greater extent than LDL, as derived from the relative amounts of apo(a) and apoB-100 that have been detected in early atherosclerotic plaques [69,70]. It seems that Lp(a) remains extracellularly due to its interactions with molecules of the extracellular matrix (ECM) and other cells [71]. The lysine binding sites of apo(a) contribute to Lp(a) hooking to ECM [72]. Indeed, transgenic mice with a mutation in KIV10 lysine binding sites display decreased amounts of Lp(a) within vessel walls [73]. A two-part interaction between Lp(a) and proteoglycan decorin has been described, comprising an electrostatic bond between apoB-100 and glycosaminoglycan (GAG) chain of decorin and a hydrophobic one between proteoglycan’s protein core and apo(a). The second interaction may explain the differences in arterial wall affinity distinguishing Lp(a) from LDL [74]. Detection of decorin in atherosclerotic arterial wall argues for a possible role of this interaction in atherogenesis [75]. Furthermore, possible ligands of Lp(a) are alpha-defensins. These proteins are neutrophil-derived, found in human atherosclerotic plaques and cluster with Lp(a). These stable complexes do not traverse the cytoplasmic membrane of the endothelial cells, and this is another mechanism that maintains Lp(a) extracellularly [76]. Nevertheless, Lp(a) is also integrated into cells, mainly in macrophages in order to form foam-cells. A multitude of interactions favor this formation. At first, there is interplay with fibronectin, a glycoprotein of the ECM. Lp(a) linked to fibronectin alone or in combination with heparin can enter macrophages and enhance lipid-driven atherogenesis [77,78,79]. In addition to this, the cholesterol content of macrophages augments internalization and degradation of Lp(a) and apo(a), and this calcium-dependent process is independent of LDL, scavenger, LRP or plasminogen receptors and is not driven by proteoglycans of the cell membrane [80]. A different mechanism demands contribution of ECM, enzymes, macrophages and vascular smooth muscle cells (VSMCs). Macrophages release sphingomyelinase that drives, in collaboration with lipoprotein lipase, adhesion of LDL and Lp(a) to bovine aortic smooth muscle cells (SMCs) and ECM. Interestingly, proteoglycan chondroitin ABC lyase attenuates this effect. SMCs do not integrate the aggregates of LDL and Lp(a), in contrast with mouse macrophages that are converted to foam cells [81]. Finally, oxidative modification of Lp(a) by malondialdehyde, in a similar way to LDL, promotes integration and degradation of this lipoprotein, mediated by the scavenger receptor of human monocyte-macrophages [82].

### 4.2. Effect of Lp(a) on Expression of Adhesion Molecules, Chemotactic Factors and Other Cytokines

Lp(a) favors initiation of atherogenesis by modulating recruitment of inflammatory cells in the vessel wall. Expression of adhesion molecules, vascular cell adhesion molecule 1 (VCAM-1) and E-selectin [83] in cultured human coronary endothelial cells [84] and intercellular adhesion molecule 1 (ICAM-1) in cultured human umbilical vein endothelial cells [85] is upregulated by Lp(a). The last effect of ICAM-1 could be partially attributed to inhibition of transforming growth factor-β (TGF-β) by Lp(a) [85]. Furthermore, Lp(a), along with β2-integrin macrophage-1 antigen (Mac-1), facilitate attachment and infiltration of monocytes. In order to achieve this, Lp(a) activates transcription factor NFkappaB, confirming inflammatory involvement in atherogenesis [86]. Another contribution of Lp(a) in orchestration of atherogenesis is the effect on chemotaxis. A study on human monocytes showed that Lp(a) exhibits chemotactic properties. The incapability of LDL to induce such responses and the inhibitory effect of plasminogen and inactivated plasmin on the Lp(a)-driven chemotaxis in monocytes suggest that the lysine binding sites of apo(a) might be responsible. The same study proposes a cGMP-dependent mechanism for this effect [87]. Lp(a) accelerates chemotaxis also indirectly by driving human endothelial cells to secrete monocyte chemotactic protein (MCP) [88]. Moreover, Lp(a) and especially the C-terminal region of apo(a) induce release of interleukin-8 (IL-8) by human THP-1 macrophages [89], promoting infiltration of neutrophils [90]. More precisely, this effect is mediated by oxidized phospholipids (OxPLs) bound to apo(a), and their interaction with apo(a) requires the lysine-binding site in KIV10 [18,91]. Except for IL-8, Lp(a) also increases expression of interleukin-1β (IL-1β) [92] and tumor necrosis factor-α (TNF-α) by macrophages, multiplying inflammation of the arterial wall [42,87,88].

### 4.3. The Impact of Oxidation in Lp(a) Function

Even though oxidized phospholipids (OxPLs) are mainly produced by oxidation of LDL, OxPLs are closely associated with Lp(a) in human circulation [93]. Low Lp(a) levels act beneficially as they remove OxPLs from the plasma [94] and induce their cleavage via a lipoprotein-associated phospholipase A2 (Lp-PLA2) mechanism [95,96]. In contrast, high Lp(a) levels result in an excessive amount of OxPLs in the arterial wall [94]. Thus, certain atheromatic actions of Lp(a) are carried out via OxPLs and include chemotaxis, formation of foam cells, enhancement of inflammation and plaque instability [93,94,97,98].

### 4.4. Effect on Vascular Smooth Muscle Cells

Lp(a) promotes proliferation of human-cultured VSMCs by blocking conversion of plasminogen to plasmin. This blockage does not enable plasmin-mediated activation of TGF-β, which is an autocrine inhibitor of SMC growth [99,100]. Additionally, apo(a) provokes a concentration-dependent chemorepulsion to SMC that is dependent on RhoA and integrin αVβ3 but unrelated to TGF-β [101].

Moreover, emerging evidence highlights the role of extracellular vesicles in pathogenesis of atherosclerosis and atherothrombosis [102,103,104]. Platelet-derived exosomes have a pivotal role in hemostasis and thrombosis because of their interaction with several vascular cell types (i.e., endothelial cells, vascular smooth muscle cells) and intercellular communication [105,106]. Finally, a single study shows that high levels of Lp(a) partially mediate SMCs and valvular interstitial cells calcification via inducing release of calcifying extracellular vesicles [107].

### 4.5. Lp(a) and Plaque Vulnerability

**Figure 3:**
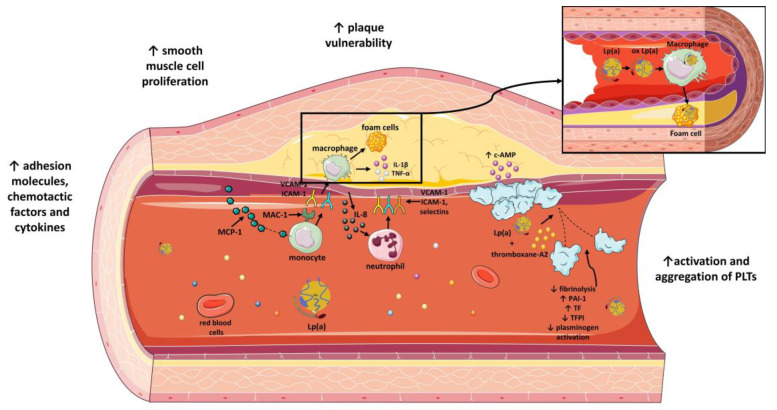
The impact of lipoprotein(a) on atherosclerotic process and atherothrombosis. Lp(a) increases atherosclerotic plaque vulnerability, vascular smooth muscle cell proliferation and adhesion of molecules, chemotactic factors and plasma cytokines. Moreover, Lp(a) enhances platelet activation and aggregation and inhibits fibrinolysis by inhibiting plasminogen activation. Lp(a) inhibits TFPI and enhances expression of TF. Moreover, Lp(a) induces increased expression of PAI-1. Lipoprotein(a): Lp(a); TFPI: tissue factor pathway inhibitor; PAI-1: plasminogen activator inhibitor-1; TF: tissue factor; ICAM-1: intercellular adhesion molecule 1; VCAM-1: vascular cell adhesion molecule 1; MAC-1: macrophage-1 antigen; MCP-1: monocyte chemoattractant protein-1; PLTs: platelets; IL-1β: interleukin 1 beta; TNF-α: tumor necrosis factor alpha; c-AMP: cyclic adenosine monophosphate; ox Lp(a): oxidized lipoprotein(a); IL-8: interleukin-8; ↓: downregulation; ↑: upregulation. Parts of the figure were drawn by using pictures from Servier Medical Art. Servier Medical Art by Servier is licensed under a Creative Commons Attribution 3.0 Unported License (https://creativecommons.org/licenses/by/3.0/) (accessed on 4 November 2022).

Lp(a) also affects the stability of atherosclerotic plaques. Metalloproteinases and elastases are enzymes, detected in atherosclerotic sites, that are responsible for the split of Lp(a) into two fragments, F1 and F2 [108,109]. F2 fragment, but not F1, is the one that interacts with fibrinogen, fibronectin and decorin, which are key molecules involved in the actions of Lp(a) [74,109]. For instance, matrix metalloproteinase-12 (MMP-12) is related to production of F1 fragment from injected human Lp(a)/apo(a) [108]. In parallel, IL-8, whose release is favored by Lp(a), downregulates expression of inhibitors of metalloproteinases [110]. Thus, a balance between Lp(a) and metalloproteinases must be achieved to avoid both the inflammatory effects of Lp(a) and the degradation of ECM by MMPs. Interestingly, Lp(a) itself also modifies plaque stability [101]. Lp(a) augments expression of micro-PAR and ICAM-1, receptors for vitronectin and fibrinogen, respectively, and thus enhances monocyte linkage to ECM. Lp(a) also upregulates expression of urokinase and urokinase receptors on monocytes and, therefore, activation of plasmin that promotes ECM shrinkage [111]. A different mechanism refers to OxPLs, as a part of apo(a), that facilitate apoptosis of endoplasmic-reticulum-stressed macrophages and, therefore, plaque necrosis [112,113]. Lp(a), thus, possesses a crucial role not only in the early phases of atherosclerosis but also later during the sequence of events that result in destabilization of atherosclerotic plaques (Figure 3).

## 5. Role of Lp(a) in Inflammation

### 5.1. Oxidized Phospholipids and Lp(a)

The reassociation between atherosclerosis and inflammation has already been documented [4]. Lp(a), as a source of free fatty acids and monoacylglycerols (via cleavage by lipoprotein lipase), can be detected in the immune system and provoke local inflammation [114]. Nevertheless, oxidative modulation of lipoproteins, including Lp(a), is a crucial mediator that can determine the evolution of inflammation. For instance, OxPLs present on OxLDLs can initiate sterile inflammation [115]. As with LDL, Lp(a), as the main carrier of OxPLs, modulates inflammation. Low Lp(a) concentrations remove OxPLs from plasma, yielding a probably beneficial effect as OxPLs can be considered as damage-associated molecular patterns (DAMPs), and, thus, the immune system is activated to restrain them [116]. Macrophage scavenger receptors gather OxPLs via Lp(a) as an antibody against apo(a) is efficient to abolish this interaction, with the lysine binding site in KIV10 being again the key mediator. Moreover, the activity of Lp(a) as platelet-activating factor acetylhydrolase (PAF-AH) [95], recently known as lipoprotein-associated phospholipase A2 (Lp-PLA2), modifies the concentration of OxPLs as they convert them to oxidized fatty acids and lysophosphatidylcholine (lysoPC), reducing their concentration. Yet, OxPLs, bound to apo(a), compete for the catalytic site of Lp-PLA2, and, as a result, attenuate its enzymatic activity and increase OxPL levels in an autocrine loop. The absence of apo(a) was found to diminish this effect, highlighting the apo(a) contribution to this effect [96]. Therefore, both beneficial and detrimental effects of Lp(a) appear to exist, related to regulation of OxPLs bioavailability in a dose-dependent manner: while an anti-inflammatory role is suggested for lower levels, this association is reversed when higher Lp(a) levels are reached [94].

### 5.2. Effect of Lp(a) in Chemotaxis

Concerning chemotaxis, Lp(a), beyond its effect on IL-8, interacts also with monocyte chemoattractant protein-1 (MCP-1), both directly through binding of OxPL with MCP-1 and indirectly by inducing its production [88,117]. Remarkably, a cGMP-driven chemotactic property of Lp(a) has been documented independent of MCP-1 and other chemokines [87]. Additionally, production of I-309, a chemotactic factor that attracts monocytes, is induced by Lp(a) in human umbilical vein endothelial cells [118]. On the other hand, experiments in apo(a) transgenic mice showed that apo(a) inhibits neutrophil recruitment due to reduced levels of neutrophil chemokines, macrophage-inflammatory protein-2, CXCL1 and CXCL2 [119,120]. Thus, Lp(a) interacts with different mediators of chemotaxis, promoting inflammation.

### 5.3. Effect of Lp(a) in Monocyte Phenotype

In vitro studies demonstrate that monocytes in the presence of Lp(a) skew towards a pro-inflammatory phenotype, exhibiting more adhesive and migratory properties. This effect was found to relate with the apo(a), especially with the site that binds OxPLs [42]. More precisely, apo(a) facilitates adhesion of monocytes on type I collagen and stimulates matrix metalloproteinase-9 (MMP-9) production and thus collagen degradation and macrophage infiltration [121]. These pro-inflammatory, M1-type macrophages, under the influence of Lp(a), release CXCL10 (IP10) chemokine in order to activate T-helper-1 (Th1) lymphocytes and natural killer (NK) cells [122]. Lp(a) also induces secretion of cytokines by macrophages, such as IL-1β, TNF-α and IL-6, multiplying, in this way, the inflammatory effect [42,123].

### 5.4. Lp(a) Induced Changes in the Inflammatory Milieu

Lp(a) was found to induce transcription factor nuclear factor-kappaB (NF-κB) in monocytes interacting with β2-integrin Mac-1, leading to augmented infiltration of monocytes [86]. Another study suggests that Lp(a) exerts this effect via autotaxin activity. Autotaxin converts lysophosphatidylcholine, a component of OxPLs, into lysophosphatidic acid, and the latter is responsible for activation of NF-κB cascade [124]. Inversely, some inflammatory cytokines can affect Lp(a) gene expression by interacting with inflammatory response elements in *LPA* gene. For instance, Il-6 increases, whereas TGF-β and TNF-α diminish, the gene expression in primary monkey hepatocyte cultures [125]. Therefore, there is a dynamic relationship between Lp(a) and inflammatory processes (Table 1).

## 6. Role of Lp(a) on Endothelial Function

### 6.1. Lp(a) Modifies the Properties of Endothelial Cells

Lp(a) can affect the transcriptional profile of endothelial cells and skew them towards a more inflammatory phenotype. Lp(a) induces expression of adhesion molecules, such as VCAM-1, E-selectin and ICAM-1, on cultured human endothelial cells [84,85]. Inhibition of TGF-beta by Lp(a) could explain in part the effect on ICAM-1 expression [85]. Notably, Lp(a) was found to accelerate senescence of endothelial cells and formation of reactive oxygen species (ROS) in human aortic endothelial cells, in combination with upregulation in expression of p53 and p21, molecules that regulate cell-cycle [149]. Moreover, ROS produced by endothelial cells in the presence of Lp(a) disrupts the integrity of the endothelial cell barrier by increasing the permeability of the endothelial cell monolayer [150]. Similarly, the apo(a) component of Lp(a) leads, via the Rho/Rho-kinase-dependent signaling pathway, to increased phosphorylation of myosin light chains and rearrangement of actin cytoskeleton, resulting in enhanced contraction of endothelial cells and loss of cell contact [151,152]. Lp(a) additionally impairs activity of endothelial progenitor cells (EPCs). Apo(a) was found to attenuate adhesion and migration properties of EPCs [153]. EPCs coexistence with Lp(a) led to a decrease in CD31-positive capillaries and did not improve ischemic limb perfusion. In vitro, generation of capillary tubes was halted by full-length recombinant apo(a) and the urinary fragments of apo(a) [154]. Apo(a) both impairs angiogenesis signaling pathways and also provokes endothelial cell apoptosis, along with modifying nuclear factors in endothelial cells via OxPLs [32]. For instance, Lp(a) activates a focal adhesion kinase-mitogen-activated protein kinase (MAPK)-dependent pathway, enhancing in that way migration and proliferation of endothelial cells [155]. Lp(a) also suppresses TGF-β, which has been found to inhibit endothelial cell migration in vitro [155,156]. Therefore, Lp(a) affects endothelial cell proliferation and permeability and also exerts anti-angiogenetic properties, interfering with different mediators.

### 6.2. Effect of Lp(a) on Endothelial-Dependent Vascular Tone

At first, Lp(a), via AKT-mediated nuclear translocation of β-catenin, leads to cyclooxygenase 2 expression in endothelial cells and release of prostaglandin E2 (PGE2), a key molecule that modulates vasodilation [157]. On the other hand, Lp(a), especially the oxidized form, reduces dose-dependently the expression of inducible nitric oxide synthase (iNOS), both at mRNA and protein levels, in lipopolysaccharide/interferon-stimulated mouse macrophages [158]. In the same regard, oxLp(a) particles, as well as oxLDL ones, were found to inhibit Ach-induced endothelium-dependent vasodilation [159]. The inhibitory effect of oxLp(a) on vasorelaxation can be multiplied by glycation. Indeed, the ox-Lp(a) and the glycated Lp(a) suppressive effects are prevented by administration of factors that act as O2 scavengers, suggesting that oxygen is responsible for the inactivation NO [160]. High Lp(a) levels affect negatively endothelium-dependent vasodilation, even in the absence of atherosclerotic lesions, suggesting a direct Lp(a) inhibitory effect or an effect mediated by increased endothelial permeability that enables direct access of acetylcholine to the VSMCs [161]. Interestingly, relatively high Lp(a) plasma levels were related to upregulated endothelium-dependent vasoconstriction in the presence of L-NMMA, a NO synthase inhibitor. A possible mechanism can be compensatory increased basal production and secretion of NO to Lp(a)-induced atherogenesis [162].

## 7. Role of Lp(a) on Thrombogenicity

The partial similarity of apo(a) with plasminogen is not enough to explain the significant role of Lp(a) in the pathways regulating hemostasis. Lp(a) affects discrete key points in primary and secondary hemostasis as well as in fibrinolysis.

### 7.1. Activation and Aggregation of PLTs

The intervention of Lp(a) in the function of platelets has already been documented since 1985 as Lp(a), via apo(a), attenuated collagen- and ADP-driven platelet aggregation and platelet production of thromboxane A2 when co-incubated with human platelets [163]. Lp(a) does not interact with platelet LDL binding sites, so it is Lp(a) that inhibits collagen- or ADP-mediated platelet aggregation [164]. There is an epitope, unique to apo(a) in Lp(a), the single arginyl-glycyl-aspartyl (RGD) epitope, which mainly mediates the suspensory effect of Lp(a) [165]. At first, Lp(a) anchors to resting platelets at a site different from the RGD epitope on the IIb subunit of the IIb/IIIa receptor [166] and then binds via the RGD epitope of apo(a) to the RGD binding site on the IIb protein of the GPIIb/IIIa receptor of agonist-stimulated platelets [165]. GPIIb/IIa is the receptor for fibrinogen, but RGD-type ligands such as apo(a) can interact with the receptor even though fibrinogen is already attached and can displace fibrinogen in an allosteric way [167]. The result is reduced collagen- and ADP-stimulated platelet aggregation [168]. Furthermore, in resting platelets, a role for cyclic-AMP has been described as low concentrations of Lp(a) in vitro induce elevation of intracellular c-AMP above basal levels and restrict collagen-stimulated platelet aggregation, whereas, at high concentrations, levels of c-AMP return to basal even though platelet aggregation continues to decline. This effect is also apo(a)-mediated [169]. Nevertheless, the role of Lp(a) and its components in activation and aggregation of PLTs has been a matter of dispute over the years. A study showed that lysine binding sites and not GPIIb/IIIa-bound fibrin affect binding of recombinant-apo(a) to platelets [170], while a later study presented that the lysine binding sites of Lp(a) are not correlated to the anti-aggregatory effect of Lp(a) in vitro [171]. The first study also exhibited a noteworthy finding: intact Lp(a) or recombinant apo(a) facilitate aggregation of platelets to subaggregant doses of arachidonic acid, whereas recombinant apo(a) does not affect the response to low doses of collagen or thrombin [170]. In line with this, native Lp(a) does not influence collagen or thrombin or ADP-driven platelet activation [172,173]. In contrast, Lp(a) was found to augment thrombin-receptor-activating hexapeptide (SFLLRN, TRAP)-induced platelet activation [173]. A different approach for the role of Lp(a) may derive from its activity as platelet-activating factor acetylhydrolase (PAF-AH) [95]. Lp(a) inhibits PAF-induced platelet activation but not via PAF-AH-action of Lp(a), and the crucial mechanism involved remains interaction with GPIIb/IIa and fibrinogen [174].

### 7.2. Interraction of Lp(a) with the Tissue Factor (TF)

Administration of Lp(a) or recombinant apo(a) (r-apo(a)) to monocytes upregulates production of TF and its presence in the cell membrane. The involvement of both the integrin αMβ2 and NFκB signaling pathways has been suggested as possible mechanisms [175].

Tissue factor pathway inhibitor is derived mainly from the endothelium and has been colocalized with vascular SMC of human atherosclerotic plaques along with apo(a) [176,177,178]. Lp(a) and apo(a) can bind to recombinant TFPI (rTFPI) in vitro in a lysine-dependent way and Lp(a) is able to impair rTFPI activity and endothelial cell surface TFPI activity in vitro independent of plasminogen [177].

### 7.3. Inhibition of Fibrinolysis by Plasmin

Apo(a) and fibrin co-localize in the arterial wall, predisposing an interaction between the two [179]. Lp(a) has an antifibrinolytic effect that may be attributed to multiple mechanisms restricting the bioavailability and action of plasmin, an enzyme that degrades fibrin. The precursor of plasmin is plasminogen, and it is significant to mention that there are two types of plasminogen: Glu-plasminogen and Lys-plasminogen, derivatives of Glu-plasminogen via plasmin-cleavage [180]. Homology with plasminogen is suggested as the most prominent linkage of Lp(a) with fibrinolysis. Lp(a) and plasminogen share similar lysine-binding sites on the fibrinogen/fibrin molecule. The size of apo(a) isoform determines inversely the affinity of Lp(a) to fibrin, with smaller size leading to greater affinity [181]. Therefore, the relative concentration of Lp(a) particles with small apo(a) isoforms can demonstrate a marker for the risk concomitant of high levels of Lp(a) [182]. Notably, plasmin catalyzes binding of Lp(a) with fibrinogen and fibrin. Therefore, a fibrin thrombus during its formation can bind with Lp(a) and simultaneously activate plasminogen, with produced plasmin further enhancing Lp(a) binding. Conversely, Lp(a) is bound to the fibrin surface, inhibiting binding of Glu-plasminogen and Lys-plasminogen to the same position and thus attenuating degradation of fibrin [183]. Moreover, reduced adherence of plasminogen to endothelial cells [184,185] and platelets can be attributed at least in part to Lp(a) [186]. Lp(a) impairs linkage of plasminogen to annexin, a plasminogen receptor of platelets and endothelial cells, and, therefore, prevents the activation of plasminogen in the surface of these cells [88]. Additionally, Lp(a) impairs the binding of t-PA to the platelet surface [174]. Moreover, factor XIIIa can catalyze linkage of Lp(a) and fibrinogen, and, remarkably, this coagulating factor co-localizes with Lp(a) in human atherosclerotic plaques [187]. This emphasizes that the interaction of Lp(a) with fibrinogen is not only dependent on the two of them but also on other factors [187]. Another and equally important mechanism described involves impairment of plasminogen activation. The effect prevails t-PA-induced activation of Glu-plasminogen and affects less t-PA activation of Lys-plasminogen and urokinase-mediated activation [188]. The initial formation of a binary complex between tPA and fibrin facilitates formation of a ternary complex between substrate (plasminogen), enzyme (tPA) and cofactor (fibrin) that precedes cleavage of plasminogen by tPA and release of plasmin [189,190]. Of note, apo(a) can regulate formation of the ternary complex as it can induce generation of a quaternary complex that includes the former. The quaternary complex is characterized by a reduced turnover number, suggesting a possible mechanism for the inhibitory effect of Lp(a) in plasminogen activation [191]. An alternative explanation may be that Lp(a) has direct crossover with t-PA [192] or Lp(a), impairing formation of the binary complex as it competes with tPA as a candidate for fibrin linkage [193]. There are also contradictory data demonstrating that Lp(a) does not interact with t-PA [194] or that apo(a) enhances instead of diminishes the bound of plasminogen with fibrin and tPA-induced plasminogen activation [195]. Finally, experiments in mice confirm the requirement of Lp(a) for t-PA-induced fibrinolysis [196]. Except for t-PA, streptokinase-induced plasminogen activation [197] is impaired, as well as urokinase, showing the broad effect of Lp(a) on fibrinolysis [188].

### 7.4. Increased Expression of Plasminogen Activator Inhibitor

Lp(a) also affects activation of plasminogen indirectly by inducing production of inhibitory molecules by surrounding cells. Expression of plasminogen activator inhibitor-1 (PAI-1) is upregulated in endothelial cells [198] in the presence of Lp(a), while monocytes from male patients with Lp(a) hyperlipidemia exhibit increased PAI-2 mRNA and protein [199].

## 8. Lp(a) and Neointimal Hyperplasia

Neointimal hyperplasia is implicated in vascular restenosis. Evidence shows that Lp(a) is correlated with vascular restenosis. Increased levels of Lp(a) were found to predict vein graft stenosis after bypass procedure [200], and apo(a) moiety has been detected in diseased vein grafts, enhancing a possible role of Lp(a) [9]. Furthermore, several studies have demonstrated an association between circulating Lp(a) and restenosis after percutaneous transluminal coronary angioplasty (PTCA). Lp(a) has been described as an independent predictor of restenosis, with higher levels correlated with higher risk of restenosis [113,201,202,203] or with an increased degree of restenosis [204]. Nevertheless, studies that reveal no significant correlation between Lp(a) and restenosis after PTCA have been developed so far [205,206].

Lp(a) may be involved in different steps of this pathophysiological mechanism. At first, Lp(a), as mentioned above, anchors to arterial wall and exhibits inflammatory properties [70]. Lp(a), in coordination with β2-integrin Mac-1, facilitates infiltration of monocytes [207], and, of note, Mac-1 blockade was found to attenuate experimental neointimal thickening [208], revealing the inflammatory character of neointimal growth. Lp(a) can also be associated with macrophages that incorporate into the injury site [209], and, indeed, it has been documented that neointimal tissue is lipid-laden [210,211], and areas of restenosis 5 years post stent implantation included cholesterol clefts, necrotizing foam cells and inflammatory cells [212]. In addition, Lp(a) increases infiltration of leukocytes by increasing expression of VCAM-1 [83].

A further step to restenosis is neointimal hyperplasia, either promoted by thrombus or by Lp(a) itself [213]. Neointimal hyperplasia is a physiologic healing response to internal or external injury to the blood vessel wall, involving all three arterial layers and characterized by proliferation of SMCs [214]. Thrombus, including platelets, mediates release or prolongs exposure to chemotactic or growth factors, such as platelet-derived growth factor, and also favors synthesis of ECM, thus preparing the local milieu and creating a “scaffold” for intimal hyperplasia [201,213,215]. Furthermore, coagulating factors such as thrombin or Xa have been proven to drive mitosis for SMCs in vitro [216,217]; thus, Lp(a) indirectly enhances neointimal hyperplasia by promoting thrombosis. Interestingly, TFPI, which, as already indicated, can be inactivated by Lp(a), may affect not only thrombosis but also SMC proliferation [177]. TFPI has been found to attenuate intimal hyperplasia post angioplasty, and this was mainly attributed to inhibition of thrombosis [218,219,220].

It is also noteworthy to mention that Lp(a) exerts direct effects on SMC proliferation. Lp(a) inhibits plasminogen activation, plasmin generation and, subsequently, production of active ΤGF-β, which impairs the migration and proliferation of SMCs [100,221]. Furthermore, when a neutralizing antibody silenced the effect of TGF-β, Lp(a), but not apo(a), induced SMC proliferation, suggesting that the LDL-particle of Lp(a) may exert also a mitogenic role [222]. Several Lp(a)-positive lesions did not contain thrombus, highlighting the significant effect of Lp(a) on neointimal growth.

## 9. Predictive Value of Lp(a) Levels

Circulating plasma Lp(a) levels increase very soon after birth, on the seventh postnatal day, and reach a constant concentration even in a few months of life [223]. Individual Lp(a) concentrations are relatively stable throughout the lifetime and range widely from <1 to >200 mg/dL in the general population [224]. Many studies suggest that women are more prone to increased Lp(a) concentrations [225,226]. According to a Danish general population cohort study (Copenhagen General Population Study), an estimated approximately 20% of the population have high concentrations corresponding to >42 mg/dL, which have long been linked to increased risk of atherosclerotic diseases [227]. Worth noting as well are the differences in Lp(a) plasma levels between different populations as Lp(a) seems to be lowest in Caucasian patients and highest in patients of African ethnicity [228].

Because >90% of circulating Lp(a) levels are genetically determined, little effect from diet and environment is detected, and concentrations over a lifespan do not vary considerably [229,230]. According to “2016 ESC/EAS Guidelines for the Management of Dyslipidaemias”, Lp(a) concentrations should be considered for Lp(a) screening in selected high-risk cases for reclassification of subjects with borderline risk, with a class IIa indication and a level of evidence: C [231], specifically subjects with a family history of premature CVD, familial hypercholesterolemia and a family history of premature CVD and/or elevated Lp(a) levels, when Lp(a) is above the 80th percentile (50 mg/dL) [6]. Moreover, patients with recurrent CVD events despite treatment for lipid-lowering and, finally, subjects with ≥5% 10-year risk of fatal CVD according to SCORE [232] should also be treated as high-risk patients, and Lp(a) levels should be examined [231]. The US National Lipid Association indicates the positive predictive power of Lp(a) measurement and provided similar recommendations on screening methods, adding in the screening procedures for patients with 10–19% Framingham risk according to the 2012 Canadian Cardiovascular Society recommendations [233,234]. Additionally, HEART UK (Hyperlipidaemia Education and Atherosclerosis Research Trust UK), in the recent consensus statement, suggests that individuals with calcific aortic valve stenosis should be evaluated for their Lp(a) levels, having employed data from the large ongoing Copenhagen General Population Study [52,235] (Table 2).

## 10. Lp(a)-Lowering Treatment

Converging evidence proclaims the role of Lp(a) in cardiovascular disease, including myocardial infarction, ischemic stroke and calcific aortic valve disease [237,238]. Its action seems to be mediated not only by its lipid-carrying content but mainly by its ability to carry and deliver oxidized phospholipids (OxPL) directly to tissue targets, which are converted to lysophosphatidic acid by autotaxin, infiltrate the endothelium and promote inflammation [238,239]. Data show the Lp(a)-lowering effect of new antilipidemic agents and establish Lp(a) as a potential new target for decreasing cardiovascular risk as lifestyle modifications are unlikely to have any effects on Lp(a) levels because of the primary genetic basis [240,241,242].

According to recent evidence, conflicting data exist regarding the effect of statins on Lp(a). Although statins remain one the most effective and safest drug category for primary prevention of ASCVD, a recent study revealed a mean 11% increase in Lp(a) levels with their use [63,243,244]. Moreover, the ILLUMINATE trial revealed that, in high-risk CVD patients, Lp(a) levels are positively and dose-dependently correlated with atorvastatin dosage [245]. Most meta-analyses on the impact of different types and dose schemas of statins show no clinically significant reduction in Lp(a) levels [246]. Despite this effect of statins, the most recent “European Atherosclerosis Society consensus statement” suggests that statin therapy should not be discontinued as their cardioprotective action overcomes any risk associated with increased Lp(a) plasma concentrations [6].

Data on the role of ezetimibe on Lp(a) circulating levels are not solid. A recent meta-analysis of seven randomized controlled trials shows an Lp(a) reduction by 7%, although this reduction is considered unable to reduce the Lp(a)-related risk of CVD events [247]. However, another large meta-analysis, collecting more robust data from 10 randomized placebo-controlled clinical trials, demonstrated that ezetimibe therapy had no effect on altering plasma Lp(a) concentrations, either as a monotherapy or in combination with a statin [248].

Niacin (nicotinic acid) has been used for over 50 years [249] for reduction in CVD events and mortality, being one of the most effective available therapies for raising HDL [250]. Niacin is also the only currently approved treatment for Lp(a) reduction, acting by silencing apo(a) gene expression in hepatocytes [247]. This effect is dose-dependent and leads to a 25% to 38% reduction in Lp(a) levels when niacin is administered at a 2 to 4 g daily dosage, respectively. However, niacin has not yet shown any effect on CVD reduction [251]. Although a large meta-analysis of 14 randomized placebo-controlled clinical trials reported a significant reduction by 23% in plasma Lp(a) concentration, the prognostic relevance of this effect has yet to be clarified [252], while the Lp(a)-lowering effect of niacin has not been linked to any clinical benefit, in terms of ASCVD events, so far [253].

Proprotein convertase subtilisin/kexin type 9 inhibitors (PCSK9i) upregulate LDL receptor (LDLR) activity [254]. Plasma PCSK9 levels are associated with Lp(a) particles in humans and mice [255]. Otherwise, in animal studies, circulating PSCK9 levels are positively associated with apo-B synthesis [256,257]. Recently, PCSK9i achieved a clinically meaningful reduction in serum Lp(a) concentrations as, in a meta-analysis of 41 studies (*n* = 64,107 randomized patients), Lp(a) levels were reduced by 26.7% [258]. The Lp(a) reduction may be attributed to reduced availability of lipoproteins containing apo-B to link with Lp(a), uptake and clearance of Lp(a) by LDLR or other hepatic receptors under a state of low LDL-C levels [257,259]. The reduction in Lp(a) under PSCK9i treatment may also be caused by reduced apo-B and Lp(a) synthesis [259]. The role of apo-B availability for Lp(a) synthesis is also supported by studies, with antisense oligonucleotides against apo-B synthesis showing parallel Lp(a) reduction [257,260]. Noteworthily, PCSK9i have proven their efficacy by decreasing incidence of acute coronary syndromes and CV deaths in patients with CAD [261], while, in experimental studies, their interaction with several proinflammatory factors is crucial [262]. Specifically, Alirocumab reduced the risk of major adverse cardiovascular events by 0.6% for each 1 mg/dL reduction in Lp(a) levels independent of LDL cholesterol reduction [261]. In the FOURIER trial, patients with high baseline Lp(a) levels experienced a greater drop in Lp(a) levels (up to 27%) after receiving evolocumab [224]. Several clinical trials have been conducted to investigate the role of PCSK9i therapy on Lp(a)-lowering [263,264,265,266,267,268]. Ge et al., in a large meta-analysis of seven clinical trials, exhibited an average reduction in Lp(a) levels of ~18%, with a follow-up period ranging from 8 to 78 weeks [269].

Recently, antisense oligonucleotides (ASO) designed to inhibit apo(a) mRNA seem promising, with preliminary results pointing to an even more substantial reduction reaching up to 90% [270]. Subcutaneously injected ASO are taken up by hepatocytes, where they bind to the apo(a)-mRNA, causing its breakdown and thus inhibiting apo(a) synthesis [271]. ISIS-APO(a)Rx, a second-generation antisense drug designed to reduce synthesis of apo(a) in liver, also shows promising results in Lp(a) reduction [272]. ISIS-APO(a)Rx reduces plasma Lp(a) in a dose-dependent manner, along with the associated OxPL. Further clinical trials are required to prove the efficacy of ISIS-APO(a)Rx to reduce CV events and calcific aortic valve stenosis [272].

## 11. Conclusions

Lp(a) is recognized as a risk factor for atherosclerotic and non-atherosclerotic cardiovascular disease, with its levels being largely genetically determined and mediated by variations in the *LPA* gene locus. Oxidized phospholipids carried by Lp(a) adversely affect various pathways (i.e., vascular inflammation, endothelial function and thrombogenicity), contributing to atherosclerosis progression. Despite the recognized role of Lp(a) in atherosclerosis, its measurement lacks a globally unified method, hampering any effort to appropriately identify individuals at higher risk. Moreover, until now, only scarce evidence exists to support the clinical benefit of Lp(a) level reduction for the few available agents. Fortunately, knowledge of the pathophysiologic mechanisms of Lp(a) synthesis and action has fueled research interest and oriented drug manufacturing efforts to more prominent approaches in management of Lp(a)-related CV risk, with novel therapeutic options anticipated in the coming years.

## Figures and Tables

**Table 1:** Studies investigating the role of lipoprotein(a) in atherosclerosis, thrombosis, inflammation and endothelial dysfunction.

Study	Study Design	Population Characteristics	Key Findings
**Atherosclerosis**			
Brown et al., 1993 [126]	Case-control	171 cases with preclinical extracranial carotid atherosclerosis and 274 control subjects free of carotid atherosclerosis.	Median levels of Lp(a) were higher in subjects with extracranial carotid atherosclerosis. An inverse correlation between apo(a) size and Lp(a) level was observed in both groups. Apo(a) phenotype distributions were similar in subjects with and without extracranial carotid atherosclerosis.
Maher et al., 1995 [127]	Randomized controlled trial	146 men aged 62 years or younger with CAD and apo B-100 levels ≥125 mg/dL. Step II Diet and lovastatin (40 mg daily) plus colestipol (30 g daily), niacin (4 g daily) plus colestipol or placebo (plus colestipol if LDL > 90th percentile) for 2.5 years.	Lp(a) was the best marker for baseline CAD severity. In patients with minimal LDL reduction (<10%), CAD progression correlated only with in-treatment Lp(a) levels, but, in patients with substantial LDL reduction (≥10%), the regression of disease correlated with LDL but not with Lp(a). Lp(a) levels were not significantly changed in either group. Patients with elevated Lp(a) (≥90th percentile) demonstrated more cardiac events (39%) under minimal LDL reduction as compared to events (9%) after substantial reduction.
Dangas et al., 1997 [128]	Cross-sectional	Coronary atheroma removed from 72 patients with stable or unstable angina.	All coronary atheroma specimens exhibited Lp(a) staining. 90% of the macrophage areas colocalized with Lp(a) positive areas, whereas 31.3% of the smooth muscle cell areas colocalized with Lp(a) positive areas. Patients with unstable angina (*n* = 46) had specimens with larger mean plaque Lp(a) areas than specimens from stable angina patients.
Gazzaruso et al., 1998 [129]	Cross-sectional	267 patients with CAD.	Lp(a) levels did not show any differences among subgroups of patients (one-vessel disease, two-vessel disease and three-or-more-vessel disease (multi-vessel). The percentage of apo(a) isoforms of low molecular weight (655 kDa) and the percentage of subjects with at least one apo(a) isoform of low molecular weight were significantly correlated with increasing number of coronary vessels stenosed.
Kronenberg et al., 1999 [130]	Prospective cohort	826 individuals (500 without carotid atherosclerosis and 326 with preexisting carotid artery disease).	Lp(a) plasma levels were significantly associated with early atherogenesis in subjects with LDL cholesterol levels above the population median (3.3 mmol/L). Apo(a) phenotype did not differ in subjects with and without early carotid atherosclerosis. Apo(a) phenotype was found a strong risk predictor of advanced atherogenesis (carotid stenosis >40%), especially LMW apo(a) phenotypes when associated with high Lp(a) plasma concentrations.
Paultre et al., 2002 [131]	Cross-sectional	Randomly selected elderly multiethnic population (173 men and 253 women, consisting of 135 African Americans, 146 Hispanics and 145 whites; mean age 70.5 ± 11.4 years).	Lp(a) levels were not associated with maximum internal carotid artery plaque thickness (MPT). In all men and white women, the amount of Lp(a) carrying the small apo(a) size was associated with carotid atherosclerosis.
Tsimikas et al., 2006 [132]	Prospective cohort	Sample from the Bruneck study (recruitment in 1990 and follow-up in 1995 and 2000 and 2005). 826 subjects had ultrasonographic follow-up of the carotid and femoral arteries in 1995 and 684 in 2000, respectively. Assessment of OxPL/apo B-100 was achieved in 765/826 subjects in 1995 and 671/684 subjects in 2000.	The OxPL/apoB and Lp(a) levels were highly correlated, and this correlation was stable through years of follow-up. The number of apo(a) KIV-2 repeats was inversely related to Lp(a) and OxPL/apoB levels. In multivariable analysis, OxPL/apoB levels were strongly and significantly associated with the presence, extent (assessed by the carotid and femoral atherosclerosis scores) and development between 1995 and 2000 of carotid and femoral atherosclerosis. OxPL/apoB were also correlated with progression of vessel stenosis >40% in the carotid arteries between 1995 and 2000 and with the presence of femoral artery stenosis. OxPL/apoB predicted the presence of symptomatic cardiovascular disease during the 10-year follow-up (ischemic stroke and transient ischemic attack, myocardial infarction and symptomatic peripheral artery disease).
Kiechl et al., 2007 [133]	Prospective cohort	Sample from the Bruneck study: 765 subjects with assessment of oxPL/apoB in 1995.	The effects of OxPL/apoB and Lp(a) were not independent of each other. OxPL/apoB levels predict 10-year CVD event rates independent of traditional risk factors, hsCRP and Framingham Risk Score.
Van der Valk et al., 2016 [42]	Cross-sectional	30 subjects with elevated Lp(a) levels (50–195 mg/dL) and 30 subjects with normal Lp(a) (2–28 mg/dL) matched for age, sex and body mass index.	Subjects with elevated Lp(a) have increased arterial inflammation and increased peripheral blood mononuclear cells trafficking to the arterial wall.
**Atherothrombosis**			
Szczeklik et al., 1992 [134]	Cross-sectional	50 healthy men aged 22–55 years divided into 2 groups: (a) with Lp(a) level > 30 mg/dL and (b) with Lp(a) < 5 mg/dL.	No relationship between serum Lp(a) with t-PA antigen and t-PA activity with PAI-1 activity was reported.
Testa et al., 1999 [135]	Cross-sectional	45 healthy subjects, 27 males and 18 females (mean age 37.54 ± 11.02 years). Intervention: different amounts of purified Lp(a) were added to a plasma with very low Lp(a) concentration (<1 mg/dL). Equal amounts of LDL were added to the plasma sample.	There was an inverse relationship between plasmin formation and the amount of added Lp(a), while no changes in plasmin formation were observed upon inclusion of LDL. Lp(a) level was negatively related to rate of plasmin formation, while plasminogen, PAI-1, t-PA and fibrinogen did not contribute significantly to this relationship.
Von Depka et al., 2000 [136]	Case-control	A total of 685 consecutive patients with at least one episode of VTE and 266 sex- and age-matched healthy controls.	Elevated Lp(a) levels (≥30 mg/dL) were found in 20% of all patients as compared to 7% among healthy controls. The coexistence of FV G1691A mutation and elevated Lp(a) was significantly more prevalent among patients with VTE than in the control group. No other established prothrombotic risk factor was found to be significantly combined with increased Lp(a).
Bilgen et al., 2005 [137]	Case-control	167 individuals: 136 CAD cases (37 with no vascular disease, 36 subjects with single vessel, 29 with double vessel and 34 with triple vessel disease) and 31 controls.	Serum Lp(a), oxidized LDL antibody (oLAB) and plasma total TFPI in patients with CAD were found to be significantly higher than in the control group. A significant positive correlation between serum Lp(a) and plasma total TFPI levels in CAD was found.
Di Nisio et al., 2005 [138]	Cross-sectional	62 outpatients (51 males, 11 females) of a clinic were included in the study with CAD history, VTE history, family history of CAD and CAD risk factors comparable among 2 groups: patients with Lp(a) above or below the median (300 mg/L).	Patients with higher Lp(a) concentrations had reduced TFPI antigen levels, whereas the TFPI activity was similar. Patients with Lp(a) below the median had statistically significant higher levels of free protein S as compared with those with higher Lp(a) values.
Undas et al., 2006 [139]	Cross-sectional	52 apparently healthy men, aged 38–63 years and 24 male survivors of MI, aged 42–65 years.	The median Lp(a) concentration was higher in patients than in healthy subjects. Participants with Lp(a) levels < 30 mg/dL had greater fibrin clot permeability than that observed in subjects with Lp(a) ≥ 30 mg/dL; either they were healthy or patients. Haplotypes with ≤22 KIV repeats and ≤8 pentanucleotide repeats were associated with high median Lp(a) levels. Subjects with ≤22 KIV repeats had lower clot permeability as compared to that observed in individuals >22 KIV repeats. These changes were detected in both patients and healthy subjects.
Pineda et al., 2009 [140]	Case-control	142 subjects presenting with MI at a young age (≤45 years) and 95 controls.	Cases had significantly higher Lp(a) levels.
Liu et al., 2022 [141]	Cross-sectional	92 subjects without statins or antiplatelet agents.	Significant correlation was found between arachidonic acid-induced average aggregation rate and ApoB, ApoA1, Lp(a), Lp-PLA2 and platelet counts.
**Inflammation and Endothelial Dysfunction**			
Wu et al., 2004 [142]	Cross-sectional	Multiethnic study of 89 healthy subjects (age 42 ± 9 years; 50 men, 39 women) free of other cardiac risk factors.	Lp(a) levels were found to correlate inversely to FMD (r = −0.33, *p* < 0.005) but not to nitrate-induced dilation. Subjects with small apo(a) size of ≤ 22 kringle IV repeats had significantly lower FMD than those with large apo(a) independent of Lp(a) levels.
Anuurad et al., 2008 [143]	Cross-sectional	167 African Americans and 259 Caucasians.	Lp(a) levels were increased among African Americans with higher vs. lower levels of hsCRP. Allele-specific apo(a) levels for medium apo(a) sizes (22–30 kringle IV repeats) were significantly higher among African Americans, with high levels of CRP or fibrinogen compared with those with low levels. No difference was found for Caucasians. No differences among African Americans or Caucasians for small apo(a) sizes.
Volpato et al., 2010 [144]	Prospective cohort	Sample from InCHIANTI study (1002 Italian subjects, 60 to 96 years of age over a 6-year follow-up with an ankle–brachial index (ABI) < 1.5. Longitudinal analysis was limited to 686 participants.	At baseline, Lp(a) level was directly related to the number of increased inflammatory markers (IL-6, IL-1 receptor antagonist, fibrinogen and CRP). Participants with elevated Lp(a) levels (≥32.9 mg/dL) demonstrated increased prevalence of lower limbs-peripheral arterial disease compared with subjects in the lowest Lp(a) quartile.
Nemati et al., 2013 [145]	Case-control	90 patients with psoriasis and 90 age-matched controls.	The levels of total cholesterol, LDL, apo B-100 and vascular adhesion protein-1 (VAP-1) in patients with psoriasis were found to be significantly higher than those of healthy subjects. In psoriatic patients, elevation of VAP-1 correlates with Lp(a) levels.
Nishino et al., 2014 [146]	Cross-sectional	441 patients with suspected vasospastic angina (VSA).	hs-CRP and Lp(a) are significantly higher in the VSA group (showed coronary spasm by the ACh test) than in the atypical chest pain group, who showed negative ACh test. Multivariate analyses identified Lp(a) as independent marker for VSA.
Mu et al., 2015 [147]	Cross-sectional	42 patients undergoing CABG (26 males and 8 females aged 48–76 years) and 12 renal artery specimens from kidney transplant donors served as controls.	Increased VCAM-1 expression in atherosclerotic patients compared with controls. In atherosclerotic patients, aortic VCAM-1 expression was positively correlated with Lp(a) (r = 0.507).
Topçiu-Shufta et al., 2016 [148]	Cross-sectional	78 patients undergoing hemodialysis treatment for a period longer than 6 months with no evidence of CVD history.	Lp(a) levels exhibit a significant positive correlation with levels of CRP, IL-6, D-dimer, fibrinogen and vWF.

Lp(a): lipoprotein (a); apo(a): apolipoprotein(a); LDL: low-density lipoprotein; CAD: coronary artery disease; apo B-100: apolipoprotein B-100; Ab: antibodies; LMW: low molecular weight; MPT: maximum internal carotid artery plaque thickness; OxPL: oxidized phospholipids; KIV-2: kringle-IV type 2; hsCRP: high-sensitivity C-reactive protein; Lp-PLA2: phospholipase A2; CVD: cardiovascular disease; t-PA: tissue plasminogen activator; PAI-1: plasminogen activator inhibitor 1; VTE: venous thromboembolism; FV: factor V; TFPI: tissue factor pathway inhibitor; MI: myocardial infarction; vWf: Von Willebrand factor; HDL: high-density lipoprotein; ApoA1: apolipoprotein A1; AMI: acute myocardial infarction; SO: surgical operations; FMD: flow-mediated dilation; IL-6: Interleukin-6; Intereukin-1; CRP: C-reactive protein; VAP-1: vascular adhesion protein-1; heFH: heterozygous familial hypercholesterolemia; VSA: vasospastic angina; Ach: acetylcholine; VCAM-1: vascular cell adhesion molecule 1.

**Table 2:** Screening of lipoprotein(a).

Lp(a) Should be Measured Once Over a Lifespan in Individuals with:
(1) premature CVD
(2) familial hypercholesterolemia, or other genetic forms of dyslipidemia
(3) recurrent CVD despite optimal lipid-lowering treatment
(4) ≥5% 10-year risk of fatal CVD according to ESC SCORE Guidelines
(5) family history of premature CVD and/or elevated Lp(a) (≥50 mg/dL)
(6) ≥10% 10-year risk of fetal CVD according to US Guidelines
(7) calcific aortic valve stenosis
(8) 10–19% Framingham risk according to the 2012 Canadian Cardiovascular Society recommendations

This table was adapted from [231,233,234,236].

## Data Availability

Not applicable.

## References

[1] Kiechl S., Willeit J. (1999). The natural course of atherosclerosis. Part I: Incidence and progression. Arterioscler. Thromb. Vasc. Biol..

[2] Schreiner P.J., Morrisett J.D., Sharrett A.R., Patsch W., Tyroler H.A., Wu K., Heiss G. (1993). Lipoprotein[a] as a risk factor for preclinical atherosclerosis. Arterioscler. Thromb. Vasc. Biol..

[3] WHO (2022). Cardiovascular Diseases.

[4] Ross R. (1999). Atherosclerosis—An inflammatory disease. N. Engl. J. Med..

[5] Lechner K., von Schacky C., McKenzie A.L., Worm N., Nixdorff U., Lechner B., Krankel N., Halle M., Krauss R.M., Scherr J. (2020). Lifestyle factors and high-risk atherosclerosis: Pathways and mechanisms beyond traditional risk factors. Eur. J. Prev. Cardiol..

[6] Kronenberg F., Mora S., Stroes E.S.G., Ference B.A., Arsenault B.J., Berglund L., Dweck M.R., Koschinsky M., Lambert G., Mach F. (2022). Lipoprotein(a) in atherosclerotic cardiovascular disease and aortic stenosis: A European Atherosclerosis Society consensus statement. Eur. Heart J..

[7] Berg K. (1963). A New Serum Type System in Man—The Lp System. Acta Pathol. Microbiol. Scand..

[8] Rath M., Niendorf A., Reblin T., Dietel M., Krebber H.J., Beisiegel U. (1989). Detection and quantification of lipoprotein(a) in the arterial wall of 107 coronary bypass patients. Arteriosclerosis.

[9] Cushing G.L., Gaubatz J.W., Nava M.L., Burdick B.J., Bocan T.M., Guyton J.R., Weilbaecher D., DeBakey M.E., Lawrie G.M., Morrisett J.D. (1989). Quantitation and localization of apolipoproteins [a] and B in coronary artery bypass vein grafts resected at re-operation. Arteriosclerosis.

[10] Kamstrup P.R., Benn M., Tybjaerg-Hansen A., Nordestgaard B.G. (2008). Extreme lipoprotein(a) levels and risk of myocardial infarction in the general population: The Copenhagen City Heart Study. Circulation.

[11] Lampsas S., Oikonomou E., Pantelidis P., Theofilis P., Grammatopoulos K., Marathonitis A., Vavuranakis M.A., Siasos G., Tousoulis D., Vavuranakis M. (2022). Lipoprotein (a) levels and Abdominal Aortic Aneurysm. A Systematic Review and Meta-analysis. Curr. Pharm. Des..

[12] Ruscica M., Sirtori C.R., Corsini A., Watts G.F., Sahebkar A. (2021). Lipoprotein(a): Knowns, unknowns and uncertainties. Pharmacol. Res..

[13] Marcovina S.M., Albers J.J. (2016). Lipoprotein (a) measurements for clinical application. J. Lipid Res..

[14] Gaubatz J.W., Heideman C., Gotto A.M., Morrisett J.D., Dahlen G.H. (1983). Human plasma lipoprotein [a]. Structural properties. J. Biol. Chem..

[15] Sommer A., Gorges R., Kostner G.M., Paltauf F., Hermetter A. (1991). Sulfhydryl-selective fluorescence labeling of lipoprotein(a) reveals evidence for one single disulfide linkage between apoproteins(a) and B-100. Biochemistry.

[16] Guevara J., Spurlino J., Jan A.Y., Yang C.Y., Tulinsky A., Prasad B.V., Gaubatz J.W., Morrisett J.D. (1993). Proposed mechanisms for binding of apo[a] kringle type 9 to apo B-100 in human lipoprotein[a]. Biophys. J..

[17] Tomlinson J.E., McLean J.W., Lawn R.M. (1989). Rhesus monkey apolipoprotein(a). Sequence, evolution, and sites of synthesis. J. Biol. Chem..

[18] Leibundgut G., Scipione C., Yin H., Schneider M., Boffa M.B., Green S., Yang X., Dennis E., Witztum J.L., Koschinsky M.L. (2013). Determinants of binding of oxidized phospholipids on apolipoprotein (a) and lipoprotein (a). J. Lipid Res..

[19] Utermann G., Weber W. (1983). Protein composition of Lp(a) lipoprotein from human plasma. FEBS Lett..

[20] Dieplinger H., Utermann G. (1999). The seventh myth of lipoprotein(a): Where and how is it assembled?. Curr. Opin. Lipidol..

[21] Koschinsky M.L., Marcovina S.M. (2004). Structure-function relationships in apolipoprotein(a): Insights into lipoprotein(a) assembly and pathogenicity. Curr. Opin. Lipidol..

[22] White A.L., Lanford R.E. (1994). Cell surface assembly of lipoprotein(a) in primary cultures of baboon hepatocytes. J. Biol. Chem..

[23] Jenner J.L., Seman L.J., Millar J.S., Lamon-Fava S., Welty F.K., Dolnikowski G.G., Marcovina S.M., Lichtenstein A.H., Barrett P.H., deLuca C. (2005). The metabolism of apolipoproteins (a) and B-100 within plasma lipoprotein (a) in human beings. Metabolism.

[24] Demant T., Seeberg K., Bedynek A., Seidel D. (2001). The metabolism of lipoprotein(a) and other apolipoprotein B-containing lipoproteins: A kinetic study in humans. Atherosclerosis.

[25] Reblin T., Rader D.J., Beisiegel U., Greten H., Brewer H.B. (1992). Correlation of apolipoprotein(a) isoproteins with Lp(a) density and distribution in fasting plasma. Atherosclerosis.

[26] Kontush A., Lindahl M., Lhomme M., Calabresi L., Chapman M.J., Davidson W.S. (2015). Structure of HDL: Particle subclasses and molecular components. High Density Lipoproteins.

[27] Ponsin G. (1988). Relationship between structure and metabolism of HDL apolipoproteins: Study with synthetic peptides. Adv. Exp. Med. Biol..

[28] Utermann G., Menzel H.J., Kraft H.G., Duba H.C., Kemmler H.G., Seitz C. (1987). Lp(a) glycoprotein phenotypes. Inheritance and relation to Lp(a)-lipoprotein concentrations in plasma. J. Clin. Investig..

[29] McLean J.W., Tomlinson J.E., Kuang W.J., Eaton D.L., Chen E.Y., Fless G.M., Scanu A.M., Lawn R.M. (1987). cDNA sequence of human apolipoprotein(a) is homologous to plasminogen. Nature.

[30] Koschinsky M.L., Beisiegel U., Henne-Bruns D., Eaton D.L., Lawn R.M. (1990). Apolipoprotein(a) size heterogeneity is related to variable number of repeat sequences in its mRNA. Biochemistry.

[31] Gabel B.R., Koschinsky M.I. (1995). Analysis of the proteolytic activity of a recombinant form of apolipoprotein(a). Biochemistry.

[32] Kalaivani V., Jaleel A. (2020). Apolipoprotein(a), an enigmatic anti-angiogenic glycoprotein in human plasma: A curse or cure?. Pharmacol. Res..

[33] Guevara J., Jan A.Y., Knapp R., Tulinsky A., Morrisett J.D. (1993). Comparison of ligand-binding sites of modeled apo[a] kringle-like sequences in human lipoprotein[a]. Arterioscler. Thromb. Vasc. Biol..

[34] Marcovina S.M., Zhang Z.H., Gaur V.P., Albers J.J. (1993). Identification of 34 apolipoprotein(a) isoforms: Differential expression of apolipoprotein(a) alleles between American blacks and whites. Biochem. Biophys. Res. Commun..

[35] Lackner C., Cohen J.C., Hobbs H.H. (1993). Molecular definition of the extreme size polymorphism in apolipoprotein(a). Hum. Mol. Genet..

[36] Marcovina S.M., Hobbs H.H., Albers J.J. (1996). Relation between number of apolipoprotein(a) kringle 4 repeats and mobility of isoforms in agarose gel: Basis for a standardized isoform nomenclature. Clin. Chem..

[37] Hixson J.E., Britten M.L., Manis G.S., Rainwater D.L. (1989). Apolipoprotein(a) (Apo(a)) glycoprotein isoforms result from size differences in Apo(a) mRNA in baboons. J. Biol. Chem..

[38] Knight B.L. (1999). Gene structure of apolipoprotein(a) and the regulation of its expression. Biochem. Soc. Trans..

[39] Zysow B.R., Lindahl G.E., Wade D.P., Knight B.L., Lawn R.M. (1995). C/T polymorphism in the 5’ untranslated region of the apolipoprotein(a) gene introduces an upstream ATG and reduces in vitro translation. Arterioscler. Thromb. Vasc. Biol..

[40] Noureen A., Fresser F., Utermann G., Schmidt K. (2015). Sequence variation within the KIV-2 copy number polymorphism of the human LPA gene in African, Asian, and European populations. PLoS ONE.

[41] Yano Y., Shimokawa K., Okada Y., Noma A. (1997). Immunolocalization of lipoprotein(a) in wounded tissues. J. Histochem. Cytochem..

[42] Van der Valk F.M., Bekkering S., Kroon J., Yeang C., Van den Bossche J., van Buul J.D., Ravandi A., Nederveen A.J., Verberne H.J., Scipione C. (2016). Oxidized Phospholipids on Lipoprotein(a) Elicit Arterial Wall Inflammation and an Inflammatory Monocyte Response in Humans. Circulation.

[43] Sabarinath P.S., Appukuttan P.S. (2015). Immunopathology of desialylation: Human plasma lipoprotein(a) and circulating anti-carbohydrate antibodies form immune complexes that recognize host cells. Mol. Cell. Biochem..

[44] Clarke R., Peden J.F., Hopewell J.C., Kyriakou T., Goel A., Heath S.C., Parish S., Barlera S., Franzosi M.G., Rust S. (2009). Genetic variants associated with Lp(a) lipoprotein level and coronary disease. N. Engl. J. Med..

[45] Erqou S., Thompson A., Di Angelantonio E., Saleheen D., Kaptoge S., Marcovina S., Danesh J. (2010). Apolipoprotein(a) isoforms and the risk of vascular disease: Systematic review of 40 studies involving 58,000 participants. J. Am. Coll. Cardiol..

[46] Zeljkovic A., Bogavac-Stanojevic N., Jelic-Ivanovic Z., Spasojevic-Kalimanovska V., Vekic J., Spasic S. (2009). Combined effects of small apolipoprotein (a) isoforms and small, dense LDL on coronary artery disease risk. Arch. Med. Res..

[47] Frank S.L., Klisak I., Sparkes R.S., Mohandas T., Tomlinson J.E., McLean J.W., Lawn R.M., Lusis A.J. (1988). The apolipoprotein(a) gene resides on human chromosome 6q26-27, in close proximity to the homologous gene for plasminogen. Hum. Genet..

[48] Utermann G. (1989). The mysteries of lipoprotein(a). Science.

[49] Erdel M., Hubalek M., Lingenhel A., Kofler K., Duba H.C., Utermann G. (1999). Counting the repetitive kringle-IV repeats in the gene encoding human apolipoprotein(a) by fibre-FISH. Nat. Genet..

[50] Sandholzer C., Hallman D.M., Saha N., Sigurdsson G., Lackner C., Csaszar A., Boerwinkle E., Utermann G. (1991). Effects of the apolipoprotein(a) size polymorphism on the lipoprotein(a) concentration in 7 ethnic groups. Hum. Genet..

[51] Coassin S., Kronenberg F. (2022). Lipoprotein(a) beyond the kringle IV repeat polymorphism: The complexity of genetic variation in the LPA gene. Atherosclerosis.

[52] Kamstrup P.R., Tybjaerg-Hansen A., Steffensen R., Nordestgaard B.G. (2009). Genetically elevated lipoprotein(a) and increased risk of myocardial infarction. JAMA.

[53] Tregouet D.A., Konig I.R., Erdmann J., Munteanu A., Braund P.S., Hall A.S., Grosshennig A., Linsel-Nitschke P., Perret C., DeSuremain M. (2009). Genome-wide haplotype association study identifies the SLC22A3-LPAL2-LPA gene cluster as a risk locus for coronary artery disease. Nat. Genet..

[54] Tsimikas S., Hall J.L. (2012). Lipoprotein(a) as a potential causal genetic risk factor of cardiovascular disease: A rationale for increased efforts to understand its pathophysiology and develop targeted therapies. J. Am. Coll. Cardiol..

[55] Kivimaki M., Magnussen C.G., Juonala M., Kahonen M., Kettunen J., Loo B.M., Lehtimaki T., Viikari J., Raitakari O.T. (2011). Conventional and Mendelian randomization analyses suggest no association between lipoprotein(a) and early atherosclerosis: The Young Finns Study. Int. J. Epidemiol..

[56] Mach F., Baigent C., Catapano A.L., Koskinas K.C., Casula M., Badimon L., Chapman M.J., De Backer G.G., Delgado V., Ference B.A. (2020). 2019 ESC/EAS Guidelines for the management of dyslipidaemias: Lipid modification to reduce cardiovascular risk. Eur. Heart J..

[57] Visseren F.L.J., Mach F., Smulders Y.M., Carballo D., Koskinas K.C., Back M., Benetos A., Biffi A., Boavida J.M., Capodanno D. (2021). 2021 ESC Guidelines on cardiovascular disease prevention in clinical practice. Eur. Heart J..

[58] Ali S., Bunker C.H., Aston C.E., Ukoli F.A., Kamboh M.I. (1998). Apolipoprotein A kringle 4 polymorphism and serum lipoprotein (a) concentrations in African blacks. Hum. Biol..

[59] Kraft H.G., Lingenhel A., Pang R.W., Delport R., Trommsdorff M., Vermaak H., Janus E.D., Utermann G. (1996). Frequency distributions of apolipoprotein(a) kringle IV repeat alleles and their effects on lipoprotein(a) levels in Caucasian, Asian, and African populations: The distribution of null alleles is non-random. Eur. J. Hum. Genet..

[60] Tsimikas S., Fazio S., Viney N.J., Xia S., Witztum J.L., Marcovina S.M. (2018). Relationship of lipoprotein(a) molar concentrations and mass according to lipoprotein(a) thresholds and apolipoprotein(a) isoform size. J. Clin. Lipidol..

[61] McConnell J.P., Guadagno P.A., Dayspring T.D., Hoefner D.M., Thiselton D.L., Warnick G.R., Harris W.S. (2014). Lipoprotein(a) mass: A massively misunderstood metric. J. Clin. Lipidol..

[62] Marcovina S.M., Navabi N., Allen S., Gonen A., Witztum J.L., Tsimikas S. (2022). Development and validation of an isoform-independent monoclonal antibody-based ELISA for measurement of lipoprotein(a). J. Lipid Res..

[63] De Boer L.M., Oorthuys A.O.J., Wiegman A., Langendam M.W., Kroon J., Spijker R., Zwinderman A.H., Hutten B.A. (2022). Statin therapy and lipoprotein(a) levels: A systematic review and meta-analysis. Eur. J. Prev. Cardiol..

[64] Guddeti R.R., Patil S., Ahmed A., Sharma A., Aboeata A., Lavie C.J., Alla V.M. (2020). Lipoprotein(a) and calcific aortic valve stenosis: A systematic review. Prog. Cardiovasc. Dis..

[65] Tsimikas S., Fazio S., Ferdinand K.C., Ginsberg H.N., Koschinsky M.L., Marcovina S.M., Moriarty P.M., Rader D.J., Remaley A.T., Reyes-Soffer G. (2018). NHLBI Working Group Recommendations to Reduce Lipoprotein(a)-Mediated Risk of Cardiovascular Disease and Aortic Stenosis. J. Am. Coll. Cardiol..

[66] Nordestgaard B.G., Tybjaerg-Hansen A., Lewis B. (1992). Influx in vivo of low density, intermediate density, and very low density lipoproteins into aortic intimas of genetically hyperlipidemic rabbits. Roles of plasma concentrations, extent of aortic lesion, and lipoprotein particle size as determinants. Arterioscler. Thromb. Vasc. Biol..

[67] Miles L.A., Fless G.M., Scanu A.M., Baynham P., Sebald M.T., Skocir P., Curtiss L.K., Levin E.G., Hoover-Plow J.L., Plow E.F. (1995). Interaction of Lp(a) with plasminogen binding sites on cells. Thromb. Haemost..

[68] Angles-Cano E., Rojas G. (2002). Apolipoprotein(a): Structure-function relationship at the lysine-binding site and plasminogen activator cleavage site. Biol. Chem..

[69] Kreuzer J., Lloyd M.B., Bok D., Fless G.M., Scanu A.M., Lusis A.J., Haberland M.E. (1994). Lipoprotein (a) displays increased accumulation compared with low-density lipoprotein in the murine arterial wall. Chem. Phys. Lipids.

[70] Smith E.B., Cochran S. (1990). Factors influencing the accumulation in fibrous plaques of lipid derived from low density lipoprotein. II. Preferential immobilization of lipoprotein (a) (Lp(a)). Atherosclerosis.

[71] van der Hoek Y.Y., Sangrar W., Cote G.P., Kastelein J.J., Koschinsky M.L. (1994). Binding of recombinant apolipoprotein(a) to extracellular matrix proteins. Arterioscler. Thromb. Vasc. Biol..

[72] Moser T.L., Enghild J.J., Pizzo S.V., Stack M.S. (1993). The extracellular matrix proteins laminin and fibronectin contain binding domains for human plasminogen and tissue plasminogen activator. J. Biol. Chem..

[73] Boonmark N.W., Lou X.J., Yang Z.J., Schwartz K., Zhang J.L., Rubin E.M., Lawn R.M. (1997). Modification of apolipoprotein(a) lysine binding site reduces atherosclerosis in transgenic mice. J. Clin. Investig..

[74] Klezovitch O., Edelstein C., Zhu L., Scanu A.M. (1998). Apolipoprotein(a) binds via its C-terminal domain to the protein core of the proteoglycan decorin. Implications for the retention of lipoprotein(a) in atherosclerotic lesions. J. Biol. Chem..

[75] Riessen R., Isner J.M., Blessing E., Loushin C., Nikol S., Wight T.N. (1994). Regional differences in the distribution of the proteoglycans biglycan and decorin in the extracellular matrix of atherosclerotic and restenotic human coronary arteries. Am. J. Pathol..

[76] Bdeir K., Cane W., Canziani G., Chaiken I., Weisel J., Koschinsky M.L., Lawn R.M., Bannerman P.G., Sachais B.S., Kuo A. (1999). Defensin promotes the binding of lipoprotein(a) to vascular matrix. Blood.

[77] Ehnholm C., Jauhiainen M., Metso J. (1990). Interaction of lipoprotein(a) with fibronectin and its potential role in atherogenesis. Eur. Heart J..

[78] Falcone D.J., Salisbury B.G. (1988). Fibronectin stimulates macrophage uptake of low density lipoprotein-heparin-collagen complexes. Arteriosclerosis.

[79] Salonen E.M., Jauhiainen M., Zardi L., Vaheri A., Ehnholm C. (1989). Lipoprotein(a) binds to fibronectin and has serine proteinase activity capable of cleaving it. EMBO J..

[80] Bottalico L.A., Keesler G.A., Fless G.M., Tabas I. (1993). Cholesterol loading of macrophages leads to marked enhancement of native lipoprotein(a) and apoprotein(a) internalization and degradation. J. Biol. Chem..

[81] Tabas I., Li Y., Brocia R.W., Xu S.W., Swenson T.L., Williams K.J. (1993). Lipoprotein lipase and sphingomyelinase synergistically enhance the association of atherogenic lipoproteins with smooth muscle cells and extracellular matrix. A possible mechanism for low density lipoprotein and lipoprotein(a) retention and macrophage foam cell formation. J. Biol. Chem..

[82] Haberland M.E., Fless G.M., Scanu A.M., Fogelman A.M. (1992). Malondialdehyde modification of lipoprotein(a) produces avid uptake by human monocyte-macrophages. J. Biol. Chem..

[83] Oikonomou E., Souvaliotis N., Lampsas S., Siasos G., Poulakou G., Theofilis P., Papaioannou T.G., Haidich A.B., Tsaousi G., Ntousopoulos V. (2022). Endothelial dysfunction in acute and long standing COVID-19: A prospective cohort study. Vascul. Pharmacol..

[84] Allen S., Khan S., Tam S., Koschinsky M., Taylor P., Yacoub M. (1998). Expression of adhesion molecules by lp(a): A potential novel mechanism for its atherogenicity. FASEB J..

[85] Takami S., Yamashita S., Kihara S., Ishigami M., Takemura K., Kume N., Kita T., Matsuzawa Y. (1998). Lipoprotein(a) enhances the expression of intercellular adhesion molecule-1 in cultured human umbilical vein endothelial cells. Circulation.

[86] Sotiriou S.N., Orlova V.V., Al-Fakhri N., Ihanus E., Economopoulou M., Isermann B., Bdeir K., Nawroth P.P., Preissner K.T., Gahmberg C.G. (2006). Lipoprotein(a) in atherosclerotic plaques recruits inflammatory cells through interaction with Mac-1 integrin. FASEB J..

[87] Syrovets T., Thillet J., Chapman M.J., Simmet T. (1997). Lipoprotein(a) is a potent chemoattractant for human peripheral monocytes. Blood.

[88] Labudovic D., Kostovska I., Tosheska Trajkovska K., Cekovska S., Brezovska Kavrakova J., Topuzovska S. (2019). Lipoprotein(a)—Link between Atherogenesis and Thrombosis. Prague Med. Rep..

[89] Klezovitch O., Edelstein C., Scanu A.M. (2001). Stimulation of interleukin-8 production in human THP-1 macrophages by apolipoprotein(a). Evidence for a critical involvement of elements in its C-terminal domain. J. Biol. Chem..

[90] Baggiolini M., Walz A., Kunkel S.L. (1989). Neutrophil-activating peptide-1/interleukin 8, a novel cytokine that activates neutrophils. J. Clin. Investig..

[91] Scipione C.A., Sayegh S.E., Romagnuolo R., Tsimikas S., Marcovina S.M., Boffa M.B., Koschinsky M.L. (2015). Mechanistic insights into Lp(a)-induced IL-8 expression: A role for oxidized phospholipid modification of apo(a). J. Lipid Res..

[92] Oikonomou E., Tsaplaris P., Anastasiou A., Xenou M., Lampsas S., Siasos G., Pantelidis P., Theofilis P., Tsatsaragkou A., Katsarou O. (2022). Interleukin-1 in Coronary Artery Disease. Curr. Top. Med. Chem..

[93] Tsimikas S., Bergmark C., Beyer R.W., Patel R., Pattison J., Miller E., Juliano J., Witztum J.L. (2003). Temporal increases in plasma markers of oxidized low-density lipoprotein strongly reflect the presence of acute coronary syndromes. J. Am. Coll. Cardiol..

[94] Bergmark C., Dewan A., Orsoni A., Merki E., Miller E.R., Shin M.J., Binder C.J., Horkko S., Krauss R.M., Chapman M.J. (2008). A novel function of lipoprotein [a] as a preferential carrier of oxidized phospholipids in human plasma. J. Lipid Res..

[95] Karabina S.A., Elisaf M.C., Goudevenos J., Siamopoulos K.C., Sideris D., Tselepis A.D. (1996). PAF-acetylhydrolase activity of Lp(a) before and during Cu(2+)-induced oxidative modification in vitro. Atherosclerosis.

[96] Tsimikas S., Tsironis L.D., Tselepis A.D. (2007). New insights into the role of lipoprotein(a)-associated lipoprotein-associated phospholipase A2 in atherosclerosis and cardiovascular disease. Arterioscler. Thromb. Vasc. Biol..

[97] Tsimikas S., Witztum J.L. (2008). The role of oxidized phospholipids in mediating lipoprotein(a) atherogenicity. Curr. Opin. Lipidol..

[98] Navab M., Hama S.Y., Reddy S.T., Ng C.J., Van Lenten B.J., Laks H., Fogelman A.M. (2002). Oxidized lipids as mediators of coronary heart disease. Curr. Opin. Lipidol..

[99] Grainger D.J., Kemp P.R., Liu A.C., Lawn R.M., Metcalfe J.C. (1994). Activation of transforming growth factor-beta is inhibited in transgenic apolipoprotein(a) mice. Nature.

[100] Grainger D.J., Kirschenlohr H.L., Metcalfe J.C., Weissberg P.L., Wade D.P., Lawn R.M. (1993). Proliferation of human smooth muscle cells promoted by lipoprotein(a). Science.

[101] Riches K., Franklin L., Maqbool A., Peckham M., Adams M., Bond J., Warburton P., Feric N.T., Koschinsky M.L., O’Regan D.J. (2013). Apolipoprotein(a) acts as a chemorepellent to human vascular smooth muscle cells via integrin alphaVbeta3 and RhoA/ROCK-mediated mechanisms. Int. J. Biochem. Cell Biol..

[102] Chang Y.J., Wang K.C. (2021). Therapeutic perspectives of extracellular vesicles and extracellular microRNAs in atherosclerosis. Curr. Top. Membr..

[103] Wang C., Li Z., Liu Y., Yuan L. (2021). Exosomes in atherosclerosis: Performers, bystanders, biomarkers, and therapeutic targets. Theranostics.

[104] Badimon L., Padro T., Arderiu G., Vilahur G., Borrell-Pages M., Suades R. (2022). Extracellular vesicles in atherothrombosis: From biomarkers and precision medicine to therapeutic targets. Immunol. Rev..

[105] Paone S., Baxter A.A., Hulett M.D., Poon I.K.H. (2019). Endothelial cell apoptosis and the role of endothelial cell-derived extracellular vesicles in the progression of atherosclerosis. Cell. Mol. Life Sci..

[106] Wang C., Liu C., Shi J., Li H., Jiang S., Zhao P., Zhang M., Du G., Fu S., Li S. (2022). Nicotine exacerbates endothelial dysfunction and drives atherosclerosis via extracellular vesicle-miRNA. Cardiovasc. Res..

[107] Rogers M.A., Atkins S.K., Zheng K.H., Singh S.A., Chelvanambi S., Pham T.H., Kuraoka S., Stroes E.S.G., Aikawa M., Aikawa E. (2022). Lipoprotein(a) Induces Vesicular Cardiovascular Calcification Revealed With Single-Extracellular Vesicle Analysis. Front. Cardiovasc. Med..

[108] Edelstein C., Shapiro S.D., Klezovitch O., Scanu A.M. (1999). Macrophage metalloelastase, MMP-12, cleaves human apolipoprotein(a) in the linker region between kringles IV-4 and IV-5. Potential relevance to lipoprotein(a) biology. J. Biol. Chem..

[109] Edelstein C., Italia J.A., Klezovitch O., Scanu A.M. (1996). Functional and metabolic differences between elastase-generated fragments of human lipoprotein[a] and apolipoprotein[a]. J. Lipid Res..

[110] Moreau M., Brocheriou I., Petit L., Ninio E., Chapman M.J., Rouis M. (1999). Interleukin-8 mediates downregulation of tissue inhibitor of metalloproteinase-1 expression in cholesterol-loaded human macrophages: Relevance to stability of atherosclerotic plaque. Circulation.

[111] Ganne F., Vasse M., Beaudeux J.L., Peynet J., Francois A., Paysant J., Lenormand B., Collet J.P., Vannier J.P., Soria J. (1999). Increased expression of u-PA and u-PAR on monocytes by LDL and Lp(a) lipoproteins—Consequences for plasmin generation and monocyte adhesion. Thromb. Haemost..

[112] Seimon T.A., Nadolski M.J., Liao X., Magallon J., Nguyen M., Feric N.T., Koschinsky M.L., Harkewicz R., Witztum J.L., Tsimikas S. (2010). Atherogenic lipids and lipoproteins trigger CD36-TLR2-dependent apoptosis in macrophages undergoing endoplasmic reticulum stress. Cell Metab..

[113] Oikonomou E., Theofilis P., Lampsas S., Katsarou O., Kalogeras K., Marinos G., Tsatsaragkou A., Anastasiou A., Lysandrou A., Gounaridi M.I. (2022). Current Concepts and Future Applications of Non-Invasive Functional and Anatomical Evaluation of Coronary Artery Disease. Life.

[114] Nordestgaard B.G. (2016). Triglyceride-Rich Lipoproteins and Atherosclerotic Cardiovascular Disease: New Insights From Epidemiology, Genetics, and Biology. Circ. Res..

[115] Stewart C.R., Stuart L.M., Wilkinson K., van Gils J.M., Deng J., Halle A., Rayner K.J., Boyer L., Zhong R., Frazier W.A. (2010). CD36 ligands promote sterile inflammation through assembly of a Toll-like receptor 4 and 6 heterodimer. Nat. Immunol..

[116] Miller Y.I., Choi S.H., Wiesner P., Fang L., Harkewicz R., Hartvigsen K., Boullier A., Gonen A., Diehl C.J., Que X. (2011). Oxidation-specific epitopes are danger-associated molecular patterns recognized by pattern recognition receptors of innate immunity. Circ. Res..

[117] Wiesner P., Tafelmeier M., Chittka D., Choi S.H., Zhang L., Byun Y.S., Almazan F., Yang X., Iqbal N., Chowdhury P. (2013). MCP-1 binds to oxidized LDL and is carried by lipoprotein(a) in human plasma. J. Lipid Res..

[118] Haque N.S., Zhang X., French D.L., Li J., Poon M., Fallon J.T., Gabel B.R., Taubman M.B., Koschinsky M., Harpel P.C. (2000). CC chemokine I-309 is the principal monocyte chemoattractant induced by apolipoprotein(a) in human vascular endothelial cells. Circulation.

[119] Hoover-Plow J., Hart E., Gong Y., Shchurin A., Schneeman T. (2009). A physiological function for apolipoprotein(a): A natural regulator of the inflammatory response. Exp. Biol. Med..

[120] Huang M., Gong Y., Grondolsky J., Hoover-Plow J. (2014). Lp(a)/apo(a) modulate MMP-9 activation and neutrophil cytokines in vivo in inflammation to regulate leukocyte recruitment. Am. J. Pathol..

[121] Sabbah N., Jaisson S., Garnotel R., Angles-Cano E., Gillery P. (2019). Small size apolipoprotein(a) isoforms enhance inflammatory and proteolytic potential of collagen-primed monocytes. Lipids Health Dis..

[122] Schmitz G., Orso E. (2015). Lipoprotein(a) hyperlipidemia as cardiovascular risk factor: Pathophysiological aspects. Clin. Res. Cardiol. Suppl..

[123] Buechler C., Ullrich H., Aslanidis C., Bared S.M., Lingenhel A., Ritter M., Schmitz G. (2003). Lipoprotein (a) downregulates lysosomal acid lipase and induces interleukin-6 in human blood monocytes. Biochim. Biophys. Acta.

[124] Bouchareb R., Mahmut A., Nsaibia M.J., Boulanger M.C., Dahou A., Lepine J.L., Laflamme M.H., Hadji F., Couture C., Trahan S. (2015). Autotaxin Derived From Lipoprotein(a) and Valve Interstitial Cells Promotes Inflammation and Mineralization of the Aortic Valve. Circulation.

[125] Ramharack R., Barkalow D., Spahr M.A. (1998). Dominant negative effect of TGF-beta1 and TNF-alpha on basal and IL-6-induced lipoprotein(a) and apolipoprotein(a) mRNA expression in primary monkey hepatocyte cultures. Arterioscler. Thromb. Vasc. Biol..

[126] Brown S.A., Morrisett J.D., Boerwinkle E., Hutchinson R., Patsch W. (1993). The relation of lipoprotein[a] concentrations and apolipoprotein[a] phenotypes with asymptomatic atherosclerosis in subjects of the Atherosclerosis Risk in Communities (ARIC) Study. Arterioscler. Thromb. Vasc. Biol..

[127] Maher V.M., Brown B.G., Marcovina S.M., Hillger L.A., Zhao X.Q., Albers J.J. (1995). Effects of lowering elevated LDL cholesterol on the cardiovascular risk of lipoprotein(a). JAMA.

[128] Dangas G., Mehran R., Harpel P.C., Sharma S.K., Marcovina S.M., Dube G., Ambrose J.A., Fallon J.T. (1998). Lipoprotein(a) and inflammation in human coronary atheroma: Association with the severity of clinical presentation. J. Am. Coll. Cardiol..

[129] Gazzaruso C., Geroldi D., Garzaniti A., Falcone C., Fratino P., Finardi G., Buscaglia P. (1998). Apolipoprotein(a) phenotypes as genetic markers of coronary atherosclerosis severity. Int. J. Cardiol..

[130] Kronenberg F., Kronenberg M.F., Kiechl S., Trenkwalder E., Santer P., Oberhollenzer F., Egger G., Utermann G., Willeit J. (1999). Role of lipoprotein(a) and apolipoprotein(a) phenotype in atherogenesis: Prospective results from the Bruneck study. Circulation.

[131] Paultre F., Tuck C.H., Boden-Albala B., Kargman D.E., Todd E., Jones J., Paik M.C., Sacco R.L., Berglund L. (2002). Relation of Apo(a) size to carotid atherosclerosis in an elderly multiethnic population. Arterioscler. Thromb. Vasc. Biol..

[132] Tsimikas S., Kiechl S., Willeit J., Mayr M., Miller E.R., Kronenberg F., Xu Q., Bergmark C., Weger S., Oberhollenzer F. (2006). Oxidized phospholipids predict the presence and progression of carotid and femoral atherosclerosis and symptomatic cardiovascular disease: Five-year prospective results from the Bruneck study. J. Am. Coll. Cardiol..

[133] Kiechl S., Willeit J., Mayr M., Viehweider B., Oberhollenzer M., Kronenberg F., Wiedermann C.J., Oberthaler S., Xu Q., Witztum J.L. (2007). Oxidized phospholipids, lipoprotein(a), lipoprotein-associated phospholipase A2 activity, and 10-year cardiovascular outcomes: Prospective results from the Bruneck study. Arterioscler. Thromb. Vasc. Biol..

[134] Szczeklik A., Radwan J., Kubicka A., Libura M., Sacha T., Swadzba J., Undas A., Szczeklik J., Jodlowski J. (1992). Plasma fibrinolytic activity in healthy subjects with high and low lipoprotein(a) concentrations. Thromb. Res..

[135] Testa R., Marcovina S.M. (1999). The rate of plasmin formation after in vitro clotting is inversely related to lipoprotein(a) plasma levels. Int. J. Clin. Lab. Res..

[136] Von Depka M., Nowak-Gottl U., Eisert R., Dieterich C., Barthels M., Scharrer I., Ganser A., Ehrenforth S. (2000). Increased lipoprotein (a) levels as an independent risk factor for venous thromboembolism. Blood.

[137] Bilgen D., Sonmez H., Ekmekci H., Ulutin T., Ozturk Z., Kokoglu E., Bayram C., Soner A., Domanic N. (2005). The relationship of TFPI, Lp(a), and oxidized LDL antibody levels in patients with coronary artery disease. Clin. Biochem..

[138] Di Nisio M., ten Wolde M., Meijers J.C., Buller H.R. (2005). Effects of high plasma lipoprotein (a) levels on tissue factor pathway inhibitor and the protein C pathway. J. Thromb. Haemost..

[139] Undas A., Stepien E., Tracz W., Szczeklik A. (2006). Lipoprotein(a) as a modifier of fibrin clot permeability and susceptibility to lysis. J. Thromb. Haemost..

[140] Pineda J., Marin F., Marco P., Roldan V., Valencia J., Ruiz-Nodar J.M., Sogorb F., Lip G.Y. (2009). Premature coronary artery disease in young (age <45) subjects: Interactions of lipid profile, thrombophilic and haemostatic markers. Int. J. Cardiol..

[141] Liu H., Fu D., Luo Y., Peng D. (2022). Independent association of Lp(a) with platelet reactivity in subjects without statins or antiplatelet agents. Sci. Rep..

[142] Wu H.D., Berglund L., Dimayuga C., Jones J., Sciacca R.R., Di Tullio M.R., Homma S. (2004). High lipoprotein(a) levels and small apolipoprotein(a) sizes are associated with endothelial dysfunction in a multiethnic cohort. J. Am. Coll. Cardiol..

[143] Anuurad E., Rubin J., Chiem A., Tracy R.P., Pearson T.A., Berglund L. (2008). High levels of inflammatory biomarkers are associated with increased allele-specific apolipoprotein(a) levels in African-Americans. J. Clin. Endocrinol. Metab..

[144] Volpato S., Vigna G.B., McDermott M.M., Cavalieri M., Maraldi C., Lauretani F., Bandinelli S., Zuliani G., Guralnik J.M., Fellin R. (2010). Lipoprotein(a), inflammation, and peripheral arterial disease in a community-based sample of older men and women (the InCHIANTI study). Am. J. Cardiol..

[145] Nemati H., Khodarahmi R., Rahmani A., Ebrahimi A., Amani M., Eftekhari K. (2013). Serum lipid profile in psoriatic patients: Correlation between vascular adhesion protein 1 and lipoprotein (a). Cell Biochem. Funct..

[146] Nishino M., Mori N., Yoshimura T., Nakamura D., Lee Y., Taniike M., Makino N., Kato H., Egami Y., Shutta R. (2014). Higher serum uric acid and lipoprotein(a) are correlated with coronary spasm. Heart Vessels.

[147] Mu W., Chen M., Gong Z., Zheng F., Xing Q. (2015). Expression of vascular cell adhesion molecule-1 in the aortic tissues of atherosclerotic patients and the associated clinical implications. Exp. Ther. Med..

[148] Topciu-Shufta V., Haxhibeqiri V., Begolli L., Baruti-Gafurri Z., Veseli S., Haxhibeqiri S., Miftari R., Kurti L., Avdiu D. (2015). Correlation of Inflammation and Lipoprotein (a) with Hypercoagulability in Hemodialysis Patients. Med. Arch..

[149] Iwabayashi M., Taniyama Y., Sanada F., Azuma J., Iekushi K., Okayama K., Chatterjee A., Rakugi H., Morishita R. (2012). Inhibition of Lp(a)-induced functional impairment of endothelial cells and endothelial progenitor cells by hepatocyte growth factor. Biochem. Biophys. Res. Commun..

[150] Wei D.H., Zhang X.L., Wang R., Zeng J.F., Zhang K., Yang J., Li S., Lin X.L., Jiang Z.S., Wang G.X. (2013). Oxidized lipoprotein(a) increases endothelial cell monolayer permeability via ROS generation. Lipids.

[151] Cho T., Jung Y., Koschinsky M.L. (2008). Apolipoprotein(a), through its strong lysine-binding site in KIV(10’), mediates increased endothelial cell contraction and permeability via a Rho/Rho kinase/MYPT1-dependent pathway. J. Biol. Chem..

[152] Pellegrino M., Furmaniak-Kazmierczak E., LeBlanc J.C., Cho T., Cao K., Marcovina S.M., Boffa M.B., Cote G.P., Koschinsky M.L. (2004). The apolipoprotein(a) component of lipoprotein(a) stimulates actin stress fiber formation and loss of cell-cell contact in cultured endothelial cells. J. Biol. Chem..

[153] Wang R., Zhang K., Li S., Tong Z., Li G., Zhao Z., Zhao Y., Liu F., Lin X., Wang Z. (2013). Apolipoprotein (a) impairs endothelial progenitor cell-mediated angiogenesis. DNA Cell Biol..

[154] Schulter V., Koolwijk P., Peters E., Frank S., Hrzenjak A., Graier W.F., van Hinsbergh V.W., Kostner G.M. (2001). Impact of apolipoprotein(a) on in vitro angiogenesis. Arterioscler. Thromb. Vasc. Biol..

[155] Liu L., Craig A.W., Meldrum H.D., Marcovina S.M., Elliott B.E., Koschinsky M.L. (2009). Apolipoprotein(a) stimulates vascular endothelial cell growth and migration and signals through integrin alphaVbeta3. Biochem. J..

[156] Muller G., Behrens J., Nussbaumer U., Bohlen P., Birchmeier W. (1987). Inhibitory action of transforming growth factor beta on endothelial cells. Proc. Natl. Acad. Sci. USA.

[157] Cho T., Romagnuolo R., Scipione C., Boffa M.B., Koschinsky M.L. (2013). Apolipoprotein(a) stimulates nuclear translocation of beta-catenin: A novel pathogenic mechanism for lipoprotein(a). Mol. Biol. Cell.

[158] Moeslinger T., Friedl R., Volf I., Brunner M., Koller E., Spieckermann P.G. (2000). Inhibition of inducible nitric oxide synthesis by oxidized lipoprotein(a) in a murine macrophage cell line. FEBS Lett..

[159] Rubanyi G.M., Freay A.D., Lawn R.M. (2000). Endothelium-dependent vasorelaxation in the aorta of transgenic mice expressing human apolipoprotein(a) or lipoprotein(a). Endothelium.

[160] Galle J., Winner B., Conzelmann E., Wanner C. (1998). Impairment of endothelial function induced by glyc-oxidized lipoprotein a [Lp(a)]. Cell. Mol. Biol..

[161] Furchgott R.F. (1983). Role of endothelium in responses of vascular smooth muscle. Circ. Res..

[162] Schlaich M.P., John S., Langenfeld M.R., Lackner K.J., Schmitz G., Schmieder R.E. (1998). Does lipoprotein(a) impair endothelial function?. J. Am. Coll. Cardiol..

[163] Barre D.E. (1998). Lipoprotein (a) reduces platelet aggregation via apo(a)-mediated decreases in thromboxane A(2)production. Platelets.

[164] Pedreno J., Fernandez R., Cullare C., Barcelo A., Elorza M.A., de Castellarnau C. (1997). Platelet integrin alpha IIb beta 3 (GPIIb-IIIa) is not implicated in the binding of LDL to intact resting platelets. Arterioscler. Thromb. Vasc. Biol..

[165] Barre D.E. (2007). Arginyl-glycyl-aspartyl (RGD) epitope of human apolipoprotein (a) inhibits platelet aggregation by antagonizing the IIb subunit of the fibrinogen (GPIIb/IIIa) receptor. Thromb. Res..

[166] Malle E., Ibovnik A., Stienmetz A., Kostner G.M., Sattler W. (1994). Identification of glycoprotein IIb as the lipoprotein(a)-binding protein on platelets. Lipoprotein(a) binding is independent of an arginyl-glycyl-aspartate tripeptide located in apolipoprotein(a). Arterioscler. Thromb. Vasc. Biol..

[167] Hu D.D., White C.A., Panzer-Knodle S., Page J.D., Nicholson N., Smith J.W. (1999). A new model of dual interacting ligand binding sites on integrin alphaIIbbeta3. J. Biol. Chem.

[168] Barre D.E. (2004). Apoprotein (A) antagonises THE GPIIB/IIIA receptor on collagen and adp-stimulated human platelets. Front. Biosci..

[169] Barre D.E. (2003). Apolipoprotein (a) mediates the lipoprotein (a)-induced biphasic shift in human platelet cyclic AMP. Thromb. Res..

[170] Martinez C., Rivera J., Loyau S., Corral J., Gonzalez-Conejero R., Lozano M.L., Vicente V., Angles-Cano E. (2001). Binding of recombinant apolipoprotein(a) to human platelets and effect on platelet aggregation. Thromb. Haemost..

[171] Barre D. (2003). Human lipoprotein (a)-induced reduction of platelet aggregation is not mediated by apolipoprotein A’s lysine-binding regions. Front. Biosci..

[172] Malle E., Ibovnik A., Leis H.J., Kostner G.M., Verhallen P.F., Sattler W. (1995). Lysine modification of LDL or lipoprotein(a) by 4-hydroxynonenal or malondialdehyde decreases platelet serotonin secretion without affecting platelet aggregability and eicosanoid formation. Arterioscler. Thromb. Vasc. Biol..

[173] Rand M.L., Sangrar W., Hancock M.A., Taylor D.M., Marcovina S.M., Packham M.A., Koschinsky M.L. (1998). Apolipoprotein(a) enhances platelet responses to the thrombin receptor-activating peptide SFLLRN. Arterioscler. Thromb. Vasc. Biol..

[174] Tsironis L.D., Mitsios J.V., Milionis H.J., Elisaf M., Tselepis A.D. (2004). Effect of lipoprotein (a) on platelet activation induced by platelet-activating factor: Role of apolipoprotein (a) and endogenous PAF-acetylhydrolase. Cardiovasc. Res..

[175] Ferretti G., Bacchetti T., Johnston T.P., Banach M., Pirro M., Sahebkar A. (2018). Lipoprotein(a): A missing culprit in the management of athero-thrombosis?. J. Cell Physiol..

[176] Bajaj M.S., Kuppuswamy M.N., Saito H., Spitzer S.G., Bajaj S.P. (1990). Cultured normal human hepatocytes do not synthesize lipoprotein-associated coagulation inhibitor: Evidence that endothelium is the principal site of its synthesis. Proc. Natl. Acad. Sci. USA.

[177] Caplice N.M., Panetta C., Peterson T.E., Kleppe L.S., Mueske C.S., Kostner G.M., Broze G.J., Simari R.D. (2001). Lipoprotein (a) binds and inactivates tissue factor pathway inhibitor: A novel link between lipoproteins and thrombosis. Blood.

[178] Broze G.J., Girard T.J. (2012). Tissue factor pathway inhibitor: Structure-function. Front. Biosci. Landmark.

[179] Beisiegel U., Niendorf A., Wolf K., Reblin T., Rath M. (1990). Lipoprotein(a) in the arterial wall. Eur. Heart J..

[180] Castellino F.J. (1984). Biochemistry of human plasminogen. Semin. Thromb. Hemost..

[181] Hervio L., Chapman M.J., Thillet J., Loyau S., Angles-Cano E. (1993). Does apolipoprotein(a) heterogeneity influence lipoprotein(a) effects on fibrinolysis?. Blood.

[182] Angles-Cano E., de la Pena Diaz A., Loyau S. (2001). Inhibition of fibrinolysis by lipoprotein(a). Ann. N. Y. Acad. Sci..

[183] Harpel P.C., Gordon B.R., Parker T.S. (1989). Plasmin catalyzes binding of lipoprotein (a) to immobilized fibrinogen and fibrin. Proc. Natl. Acad. Sci. USA.

[184] Miles L.A., Fless G.M., Levin E.G., Scanu A.M., Plow E.F. (1989). A potential basis for the thrombotic risks associated with lipoprotein(a). Nature.

[185] Hajjar K.A., Gavish D., Breslow J.L., Nachman R.L. (1989). Lipoprotein(a) modulation of endothelial cell surface fibrinolysis and its potential role in atherosclerosis. Nature.

[186] Ezratty A., Simon D.I., Loscalzo J. (1993). Lipoprotein(a) binds to human platelets and attenuates plasminogen binding and activation. Biochemistry.

[187] Romanic A.M., Arleth A.J., Willette R.N., Ohlstein E.H. (1998). Factor XIIIa cross-links lipoprotein(a) with fibrinogen and is present in human atherosclerotic lesions. Circ. Res..

[188] Sangrar W., Bajzar L., Nesheim M.E., Koschinsky M.L. (1995). Antifibrinolytic effect of recombinant apolipoprotein(a) in vitro is primarily due to attenuation of tPA-mediated Glu-plasminogen activation. Biochemistry.

[189] Hoylaerts M., Rijken D.C., Lijnen H.R., Collen D. (1982). Kinetics of the activation of plasminogen by human tissue plasminogen activator. Role of fibrin. J. Biol. Chem..

[190] Horrevoets A.J., Pannekoek H., Nesheim M.E. (1997). A steady-state template model that describes the kinetics of fibrin-stimulated [Glu1]- and [Lys78]plasminogen activation by native tissue-type plasminogen activator and variants that lack either the finger or kringle-2 domain. J. Biol. Chem..

[191] Hancock M.A., Boffa M.B., Marcovina S.M., Nesheim M.E., Koschinsky M.L. (2003). Inhibition of plasminogen activation by lipoprotein(a): Critical domains in apolipoprotein(a) and mechanism of inhibition on fibrin and degraded fibrin surfaces. J. Biol. Chem..

[192] Simon D.I., Fless G.M., Scanu A.M., Loscalzo J. (1991). Tissue-type plasminogen activator binds to and is inhibited by surface-bound lipoprotein(a) and low-density lipoprotein. Biochemistry.

[193] Loscalzo J., Weinfeld M., Fless G.M., Scanu A.M. (1990). Lipoprotein(a), fibrin binding, and plasminogen activation. Arteriosclerosis.

[194] Hajjar K.A., Hamel N.M., Harpel P.C., Nachman R.L. (1987). Binding of tissue plasminogen activator to cultured human endothelial cells. J. Clin. Investig..

[195] Liu J.N., Harpel P.C., Pannell R., Gurewich V. (1993). Lipoprotein(a): A kinetic study of its influence on fibrin-dependent plasminogen activation by prourokinase or tissue plasminogen activator. Biochemistry.

[196] Palabrica T.M., Liu A.C., Aronovitz M.J., Furie B., Lawn R.M., Furie B.C. (1995). Antifibrinolytic activity of apolipoprotein(a) in vivo: Human apolipoprotein(a) transgenic mice are resistant to tissue plasminogen activator-mediated thrombolysis. Nat. Med..

[197] Edelberg J.M., Gonzalez-Gronow M., Pizzo S.V. (1989). Lipoprotein a inhibits streptokinase-mediated activation of human plasminogen. Biochemistry.

[198] Etingin O.R., Hajjar D.P., Hajjar K.A., Harpel P.C., Nachman R.L. (1991). Lipoprotein (a) regulates plasminogen activator inhibitor-1 expression in endothelial cells. A potential mechanism in thrombogenesis. J. Biol. Chem..

[199] Buechler C., Ullrich H., Ritter M., Porsch-Oezcueruemez M., Lackner K.J., Barlage S., Friedrich S.O., Kostner G.M., Schmitz G. (2001). Lipoprotein (a) up-regulates the expression of the plasminogen activator inhibitor 2 in human blood monocytes. Blood.

[200] Hoff H.F., Beck G.J., Skibinski C.I., Jurgens G., O’Neil J., Kramer J., Lytle B. (1988). Serum Lp(a) level as a predictor of vein graft stenosis after coronary artery bypass surgery in patients. Circulation.

[201] Hearn J.A., Donohue B.C., Ba’albaki H., Douglas J.S., King S.B., Lembo N.J., Roubin G.S., Sgoutas D.S. (1992). Usefulness of serum lipoprotein (a) as a predictor of restenosis after percutaneous transluminal coronary angioplasty. Am. J. Cardiol..

[202] Desmarais R.L., Sarembock I.J., Ayers C.R., Vernon S.M., Powers E.R., Gimple L.W. (1995). Elevated serum lipoprotein(a) is a risk factor for clinical recurrence after coronary balloon angioplasty. Circulation.

[203] Kamitani T., Taniguchi T., Miyai N., Kawasaki T., Kawasaki S., Sugihara H. (2005). Association between plasma lipoprotein(a) concentration and restenosis after stent implantation. Circ. J..

[204] Tenda K., Saikawa T., Maeda T., Sato Y., Niwa H., Inoue T., Yonemochi H., Maruyama T., Shimoyama N., Aragaki S. (1993). The relationship between serum lipoprotein(a) and restenosis after initial elective percutaneous transluminal coronary angioplasty. Jpn. Circ. J..

[205] Shah P.K., Amin J. (1992). Low high density lipoprotein level is associated with increased restenosis rate after coronary angioplasty. Circulation.

[206] Cooke T., Sheahan R., Foley D., Reilly M., D’Arcy G., Jauch W., Gibney M., Gearty G., Crean P., Walsh M. (1994). Lipoprotein(a) in restenosis after percutaneous transluminal coronary angioplasty and coronary artery disease. Circulation.

[207] Nordestgaard B.G., Chapman M.J., Ray K., Boren J., Andreotti F., Watts G.F., Ginsberg H., Amarenco P., Catapano A., Descamps O.S. (2010). Lipoprotein(a) as a cardiovascular risk factor: Current status. Eur. Heart J..

[208] Rogers C., Edelman E.R., Simon D.I. (1998). A mAb to the beta2-leukocyte integrin Mac-1 (CD11b/CD18) reduces intimal thickening after angioplasty or stent implantation in rabbits. Proc. Natl. Acad. Sci. USA.

[209] Zioncheck T.F., Powell L.M., Rice G.C., Eaton D.L., Lawn R.M. (1991). Interaction of recombinant apolipoprotein(a) and lipoprotein(a) with macrophages. J. Clin. Investig..

[210] Araki T., Nakamura M., Sugi K. (2014). Characterization of in-stent neointimal tissue components following drug-eluting stent implantation according to the phase of restenosis using a 40-MHz intravascular ultrasound imaging system. J. Cardiol..

[211] Takano M., Yamamoto M., Inami S., Murakami D., Ohba T., Seino Y., Mizuno K. (2009). Appearance of lipid-laden intima and neovascularization after implantation of bare-metal stents extended late-phase observation by intracoronary optical coherence tomography. J. Am. Coll. Cardiol..

[212] Hasegawa K., Tamai H., Kyo E., Kosuga K., Ikeguchi S., Hata T., Okada M., Fujita S., Tsuji T., Takeda S. (2006). Histopathological findings of new in-stent lesions developed beyond five years. Catheter Cardiovasc. Interv..

[213] Ross R., Glomset J., Kariya B., Harker L. (1974). A platelet-dependent serum factor that stimulates the proliferation of arterial smooth muscle cells in vitro. Proc. Natl. Acad. Sci. USA.

[214] Braga S.F., Neves J.R., Ferreira J., Carrilho C., Simoes J.C., Mesquita A. (2019). Neointimal Hyperplasia. Rev. Port. Cir. Cardiotorac. Vasc..

[215] Schwartz R.S., Holmes D.R., Topol E.J. (1992). The restenosis paradigm revisited: An alternative proposal for cellular mechanisms. J. Am. Coll. Cardiol..

[216] McNamara C.A., Sarembock I.J., Gimple L.W., Fenton J.W., Coughlin S.R., Owens G.K. (1993). Thrombin stimulates proliferation of cultured rat aortic smooth muscle cells by a proteolytically activated receptor. J. Clin. Investig..

[217] Gasic G.P., Arenas C.P., Gasic T.B., Gasic G.J. (1992). Coagulation factors X, Xa, and protein S as potent mitogens of cultured aortic smooth muscle cells. Proc. Natl. Acad. Sci. USA.

[218] Oltrona L., Speidel C.M., Recchia D., Wickline S.A., Eisenberg P.R., Abendschein D.R. (1997). Inhibition of tissue factor-mediated coagulation markedly attenuates stenosis after balloon-induced arterial injury in minipigs. Circulation.

[219] Jang Y., Guzman L.A., Lincoff A.M., Gottsauner-Wolf M., Forudi F., Hart C.E., Courtman D.W., Ezban M., Ellis S.G., Topol E.J. (1995). Influence of blockade at specific levels of the coagulation cascade on restenosis in a rabbit atherosclerotic femoral artery injury model. Circulation.

[220] Brown D.M., Kania N.M., Choi E.T., Lantieri L.A., Pasia E.N., Wun T.C., Khouri R.K. (1996). Local irrigation with tissue factor pathway inhibitor inhibits intimal hyperplasia induced by arterial interventions. Arch. Surg..

[221] Kojima S., Harpel P.C., Rifkin D.B. (1991). Lipoprotein (a) inhibits the generation of transforming growth factor beta: An endogenous inhibitor of smooth muscle cell migration. J. Cell Biol..

[222] Miyata M., Biro S., Kaieda H., Tanaka H. (1995). Lipoprotein(a) stimulates the proliferation of cultured human arterial smooth muscle cells through two pathways. FEBS Lett..

[223] Strandkjaer N., Hansen M.K., Nielsen S.T., Frikke-Schmidt R., Tybjaerg-Hansen A., Nordestgaard B.G., Tabor A., Bundgaard H., Iversen K., Kamstrup P.R. (2022). Lipoprotein(a) Levels at Birth and in Early Childhood: The COMPARE Study. J. Clin. Endocrinol. Metab..

[224] O’Donoghue M.L., Fazio S., Giugliano R.P., Stroes E.S.G., Kanevsky E., Gouni-Berthold I., Im K., Lira Pineda A., Wasserman S.M., Ceska R. (2019). Lipoprotein(a), PCSK9 Inhibition, and Cardiovascular Risk. Circulation.

[225] Frohlich J., Dobiasova M., Adler L., Francis M. (2004). Gender differences in plasma levels of lipoprotein (a) in patients with angiographically proven coronary artery disease. Physiol. Res..

[226] Nago N., Kayaba K., Hiraoka J., Matsuo H., Goto T., Kario K., Tsutsumi A., Nakamura Y., Igarashi M. (1995). Lipoprotein(a) levels in the Japanese population: Influence of age and sex, and relation to atherosclerotic risk factors. The Jichi Medical School Cohort Study. Am. J. Epidemiol..

[227] Simony S.B., Mortensen M.B., Langsted A., Afzal S., Kamstrup P.R., Nordestgaard B.G. (2022). Sex differences of lipoprotein(a) levels and associated risk of morbidity and mortality by age: The Copenhagen General Population Study. Atherosclerosis.

[228] Enkhmaa B., Anuurad E., Berglund L. (2016). Lipoprotein (a): Impact by ethnicity and environmental and medical conditions. J. Lipid Res..

[229] Corsetti J.P., Sterry J.A., Sparks J.D., Sparks C.E., Weintraub M. (1991). Effect of weight loss on serum lipoprotein(a) concentrations in an obese population. Clin. Chem..

[230] Jenner J.L., Ordovas J.M., Lamon-Fava S., Schaefer M.M., Wilson P.W., Castelli W.P., Schaefer E.J. (1993). Effects of age, sex, and menopausal status on plasma lipoprotein(a) levels. The Framingham Offspring Study. Circulation.

[231] Catapano A.L., Graham I., De Backer G., Wiklund O., Chapman M.J., Drexel H., Hoes A.W., Jennings C.S., Landmesser U., Pedersen T.R. (2016). 2016 ESC/EAS Guidelines for the Management of Dyslipidaemias. Eur. Heart J..

[232] Conroy R.M., Pyorala K., Fitzgerald A.P., Sans S., Menotti A., De Backer G., De Bacquer D., Ducimetiere P., Jousilahti P., Keil U. (2003). Estimation of ten-year risk of fatal cardiovascular disease in Europe: The SCORE project. Eur. Heart J..

[233] Davidson M.H., Ballantyne C.M., Jacobson T.A., Bittner V.A., Braun L.T., Brown A.S., Brown W.V., Cromwell W.C., Goldberg R.B., McKenney J.M. (2011). Clinical utility of inflammatory markers and advanced lipoprotein testing: Advice from an expert panel of lipid specialists. J. Clin. Lipidol..

[234] Anderson T.J., Gregoire J., Hegele R.A., Couture P., Mancini G.B., McPherson R., Francis G.A., Poirier P., Lau D.C., Grover S. (2013). 2012 update of the Canadian Cardiovascular Society guidelines for the diagnosis and treatment of dyslipidemia for the prevention of cardiovascular disease in the adult. Can. J. Cardiol..

[235] Cegla J., Neely R.D.G., France M., Ferns G., Byrne C.D., Halcox J., Datta D., Capps N., Shoulders C., Qureshi N. (2019). HEART UK consensus statement on Lipoprotein(a): A call to action. Atherosclerosis.

[236] Cegla J., France M., Marcovina S.M., Neely R.D.G. (2021). Lp(a): When and how to measure it. Ann. Clin. Biochem..

[237] Nissen S.E., Wolski K., Cho L., Nicholls S.J., Kastelein J., Leitersdorf E., Landmesser U., Blaha M., Lincoff A.M., Morishita R. (2022). Lipoprotein(a) levels in a global population with established atherosclerotic cardiovascular disease. Open Heart.

[238] Farnier M., Chague F., Maza M., Bichat F., Masson D., Cottin Y., Zeller M. (2022). High lipoprotein(a) levels predict severity of coronary artery disease in patients hospitalized for acute myocardial infarction. Data from the French RICO survey. J. Clin. Lipidol..

[239] Clarke R., Hammami I., Sherliker P., Valdes-Marquez E., Watkins H., Hill M., Yang X., Tsimikas S., Hopewell J.C., Consortium P. (2022). Oxidized phospholipids on apolipoprotein B-100 versus plasminogen and risk of coronary heart disease in the PROCARDIS study. Atherosclerosis.

[240] Nurmohamed N.S., Kaiser Y., Schuitema P.C.E., Ibrahim S., Nierman M., Fischer J.C., Chamuleau S.A.J., Knaapen P., Stroes E.S.G. (2022). Finding very high lipoprotein(a): The need for routine assessment. Eur. J. Prev. Cardiol..

[241] Khera A.V., Everett B.M., Caulfield M.P., Hantash F.M., Wohlgemuth J., Ridker P.M., Mora S. (2014). Lipoprotein(a) concentrations, rosuvastatin therapy, and residual vascular risk: An analysis from the JUPITER Trial (Justification for the Use of Statins in Prevention: An Intervention Trial Evaluating Rosuvastatin). Circulation.

[242] Madsen C.M., Kamstrup P.R., Langsted A., Varbo A., Nordestgaard B.G. (2020). Lipoprotein(a)-Lowering by 50 mg/dL (105 nmol/L) May Be Needed to Reduce Cardiovascular Disease 20% in Secondary Prevention: A Population-Based Study. Arterioscler. Thromb. Vasc. Biol..

[243] Willeit P., Ridker P.M., Nestel P.J., Simes J., Tonkin A.M., Pedersen T.R., Schwartz G.G., Olsson A.G., Colhoun H.M., Kronenberg F. (2018). Baseline and on-statin treatment lipoprotein(a) levels for prediction of cardiovascular events: Individual patient-data meta-analysis of statin outcome trials. Lancet.

[244] Tsimikas S., Gordts P., Nora C., Yeang C., Witztum J.L. (2020). Statin therapy increases lipoprotein(a) levels. Eur. Heart J..

[245] Arsenault B.J., Petrides F., Tabet F., Bao W., Hovingh G.K., Boekholdt S.M., Ramin-Mangata S., Meilhac O., DeMicco D., Rye K.A. (2018). Effect of atorvastatin, cholesterol ester transfer protein inhibition, and diabetes mellitus on circulating proprotein subtilisin kexin type 9 and lipoprotein(a) levels in patients at high cardiovascular risk. J. Clin. Lipidol..

[246] Wang X., Li J., Ju J., Fan Y., Xu H. (2021). Effect of different types and dosages of statins on plasma lipoprotein(a) levels: A network meta-analysis. Pharmacol. Res..

[247] Awad K., Mikhailidis D.P., Katsiki N., Muntner P., Banach M., Lipid and Blood Pressure Meta-Analysis Collaboration Group (2018). Effect of Ezetimibe Monotherapy on Plasma Lipoprotein(a) Concentrations in Patients with Primary Hypercholesterolemia: A Systematic Review and Meta-Analysis of Randomized Controlled Trials. Drugs.

[248] Sahebkar A., Simental-Mendia L.E., Pirro M., Banach M., Watts G.F., Sirtori C., Al-Rasadi K., Atkin S.L. (2018). Impact of ezetimibe on plasma lipoprotein(a) concentrations as monotherapy or in combination with statins: A systematic review and meta-analysis of randomized controlled trials. Sci. Rep..

[249] Altschul R., Hoffer A., Stephen J.D. (1955). Influence of nicotinic acid on serum cholesterol in man. Arch. Biochem. Biophys..

[250] Guyton J.R., Blazing M.A., Hagar J., Kashyap M.L., Knopp R.H., McKenney J.M., Nash D.T., Nash S.D. (2000). Extended-release niacin vs gemfibrozil for the treatment of low levels of high-density lipoprotein cholesterol. Niaspan-Gemfibrozil Study Group. Arch. Intern. Med..

[251] Parish S., Hopewell J.C., Hill M.R., Marcovina S., Valdes-Marquez E., Haynes R., Offer A., Pedersen T.R., Baigent C., Collins R. (2018). Impact of Apolipoprotein(a) Isoform Size on Lipoprotein(a) Lowering in the HPS2-THRIVE Study. Circ. Genom. Precis. Med..

[252] Sahebkar A., Reiner Z., Simental-Mendia L.E., Ferretti G., Cicero A.F. (2016). Effect of extended-release niacin on plasma lipoprotein(a) levels: A systematic review and meta-analysis of randomized placebo-controlled trials. Metabolism.

[253] Julius U., Fischer S. (2013). Nicotinic acid as a lipid-modifying drug—A review. Atheroscler. Suppl..

[254] Reyes-Soffer G., Pavlyha M., Ngai C., Thomas T., Holleran S., Ramakrishnan R., Karmally W., Nandakumar R., Fontanez N., Obunike J. (2017). Effects of PCSK9 Inhibition With Alirocumab on Lipoprotein Metabolism in Healthy Humans. Circulation.

[255] Tavori H., Christian D., Minnier J., Plubell D., Shapiro M.D., Yeang C., Giunzioni I., Croyal M., Duell P.B., Lambert G. (2016). PCSK9 Association With Lipoprotein(a). Circ. Res..

[256] Stein E.A., Raal F.J. (2013). Insights into PCSK9, low-density lipoprotein receptor, and low-density lipoprotein cholesterol metabolism: Of mice and man. Circulation.

[257] Sun H., Samarghandi A., Zhang N., Yao Z., Xiong M., Teng B.B. (2012). Proprotein convertase subtilisin/kexin type 9 interacts with apolipoprotein B and prevents its intracellular degradation, irrespective of the low-density lipoprotein receptor. Arterioscler. Thromb. Vasc. Biol..

[258] Farmakis I., Doundoulakis I., Pagiantza A., Zafeiropoulos S., Antza C., Karvounis H., Giannakoulas G. (2021). Lipoprotein(a) Reduction With Proprotein Convertase Subtilisin/Kexin Type 9 Inhibitors: A Systematic Review and Meta-analysis. J. Cardiovasc. Pharmacol..

[259] Romagnuolo R., Scipione C.A., Boffa M.B., Marcovina S.M., Seidah N.G., Koschinsky M.L. (2015). Lipoprotein(a) catabolism is regulated by proprotein convertase subtilisin/kexin type 9 through the low density lipoprotein receptor. J. Biol. Chem..

[260] Villard E.F., Thedrez A., Blankenstein J., Croyal M., Tran T.T., Poirier B., Le Bail J.C., Illiano S., Nobecourt E., Krempf M. (2016). PCSK9 Modulates the Secretion But Not the Cellular Uptake of Lipoprotein(a) Ex Vivo: An Effect Blunted by Alirocumab. JACC Basic Transl. Sci..

[261] Bittner V.A., Szarek M., Aylward P.E., Bhatt D.L., Diaz R., Edelberg J.M., Fras Z., Goodman S.G., Halvorsen S., Hanotin C. (2020). Effect of Alirocumab on Lipoprotein(a) and Cardiovascular Risk After Acute Coronary Syndrome. J. Am. Coll. Cardiol..

[262] Greco M.F., Rizzuto A.S., Zara M., Cafora M., Favero C., Solazzo G., Giusti I., Adorni M.P., Zimetti F., Dolo V. (2022). PCSK9 Confers Inflammatory Properties to Extracellular Vesicles Released by Vascular Smooth Muscle Cells. Int. J. Mol. Sci..

[263] Moriarty P.M., Parhofer K.G., Babirak S.P., Cornier M.A., Duell P.B., Hohenstein B., Leebmann J., Ramlow W., Schettler V., Simha V. (2016). Alirocumab in patients with heterozygous familial hypercholesterolaemia undergoing lipoprotein apheresis: The ODYSSEY ESCAPE trial. Eur. Heart J..

[264] Stein E.A., Gipe D., Bergeron J., Gaudet D., Weiss R., Dufour R., Wu R., Pordy R. (2012). Effect of a monoclonal antibody to PCSK9, REGN727/SAR236553, to reduce low-density lipoprotein cholesterol in patients with heterozygous familial hypercholesterolaemia on stable statin dose with or without ezetimibe therapy: A phase 2 randomised controlled trial. Lancet.

[265] Ginsberg H.N., Rader D.J., Raal F.J., Guyton J.R., Baccara-Dinet M.T., Lorenzato C., Pordy R., Stroes E. (2016). Efficacy and Safety of Alirocumab in Patients with Heterozygous Familial Hypercholesterolemia and LDL-C of 160 mg/dl or Higher. Cardiovasc. Drugs Ther..

[266] Raal F.J., Stein E.A., Dufour R., Turner T., Civeira F., Burgess L., Langslet G., Scott R., Olsson A.G., Sullivan D. (2015). PCSK9 inhibition with evolocumab (AMG 145) in heterozygous familial hypercholesterolaemia (RUTHERFORD-2): A randomised, double-blind, placebo-controlled trial. Lancet.

[267] Raal F., Scott R., Somaratne R., Bridges I., Li G., Wasserman S.M., Stein E.A. (2012). Low-density lipoprotein cholesterol-lowering effects of AMG 145, a monoclonal antibody to proprotein convertase subtilisin/kexin type 9 serine protease in patients with heterozygous familial hypercholesterolemia: The Reduction of LDL-C with PCSK9 Inhibition in Heterozygous Familial Hypercholesterolemia Disorder (RUTHERFORD) randomized trial. Circulation.

[268] Raal F.J., Honarpour N., Blom D.J., Hovingh G.K., Xu F., Scott R., Wasserman S.M., Stein E.A., Investigators T. (2015). Inhibition of PCSK9 with evolocumab in homozygous familial hypercholesterolaemia (TESLA Part B): A randomised, double-blind, placebo-controlled trial. Lancet.

[269] Ge X., Zhu T., Zeng H., Yu X., Li J., Xie S., Wan J., Yang H., Huang K., Zhang W. (2021). A Systematic Review and Meta-Analysis of Therapeutic Efficacy and Safety of Alirocumab and Evolocumab on Familial Hypercholesterolemia. Biomed Res. Int..

[270] Borrelli M.J., Youssef A., Boffa M.B., Koschinsky M.L. (2019). New Frontiers in Lp(a)-Targeted Therapies. Trends Pharmacol. Sci..

[271] Graham M.J., Viney N., Crooke R.M., Tsimikas S. (2016). Antisense inhibition of apolipoprotein (a) to lower plasma lipoprotein (a) levels in humans. J. Lipid Res..

[272] Tsimikas S., Viney N.J., Hughes S.G., Singleton W., Graham M.J., Baker B.F., Burkey J.L., Yang Q., Marcovina S.M., Geary R.S. (2015). Antisense therapy targeting apolipoprotein(a): A randomised, double-blind, placebo-controlled phase 1 study. Lancet.

